# What influences attitudes about artificial intelligence adoption: Evidence from U.S. local officials

**DOI:** 10.1371/journal.pone.0257732

**Published:** 2021-10-20

**Authors:** Michael C. Horowitz, Lauren Kahn

**Affiliations:** 1 Perry World House, University of Pennsylvania, Philadelphia, Pennsylvania, United States of America; 2 Council on Foreign Relations, Washington, D.C., United States of America; Bucharest University of Economic Studies, ROMANIA

## Abstract

Rapid advances in machine learning and related techniques have increased optimism about self-driving cars, autonomous surgery, and other uses of artificial intelligence (AI). But adoption of these technologies is not simply a matter of breakthroughs in the design and training of algorithms. Regulators around the world will have to make a litany of choices about law and policy surrounding AI. To advance knowledge of how they will make these choices, we draw on a unique survey pool—690 local officials in the United States—a representative sample of U.S. local officials. These officials will make many of the decisions about AI adoption, from government use to regulation, given the decentralized structure of the United States. The results show larger levels of support for autonomous vehicles than autonomous surgery. Moreover, those that used ridesharing apps prior to the COVID-19 pandemic are significantly more supportive of autonomous vehicles. We also find that self-reported familiarity with AI is correlated with increased approval of AI uses in a variety of areas, including facial recognition, natural disaster impact planning, and even military surveillance. Related, those who expressed greater opposition to AI adoption also appear more concerned about trade-offs between privacy and information and bias in algorithms. Finally, the explanatory logic used by respondents varies based on gender and prior experience with AI, which we demonstrate with quantitative text analysis.

## Introduction

Rapid advances in machine learning and related techniques have increased optimism that the epoch of self-driving cars, robotic surgery, and other uses of autonomous systems enabled by artificial intelligence (AI) may nearly be upon us. As Fei Fei Li notes, “AI and the fourth industrial revolution will impact every aspect of people’s lives” [1]. However, the development and implementation of these technologies will not be seamless nor without hesitancy.

As with any new technology, the adoption of AI-enabled technologies will become more likely if the average person’s concerns are addressed. While expectations of how technology will perform play a key role in determining receptivity—and therefore the ease and speed with which technology will be adopted—reliability, security, and privacy also condition attitudes about technology that undoubtedly impact their adoption timelines [2–4, 25]. In turn, adoption processes inform how technologies shape the world in practice [5]. When looking at AI adoption, in particular, the attitudes of the general public, policymakers, governments, and standard-setting organizations tasked with making a litany of regulatory choices about when and how to allow the use of different applications of artificial intelligence, will be critical.

Moreover, elites are already facing decisions about how AI will be implemented in general, and within government. For example, a debate has emerged surrounding local police uses of AI for surveillance in the United States. Concerns about bias, privacy, and related issues are being raised by the public and substantiated by empirical evidence and testing [6, 7].

The attitudes of public officials matter not just because they will make key regulatory decisions, but because they help shape the beliefs of the general public. The average citizen cannot follow all policy issues, so seeks guidance by looking at cues from elites they perceive as relevant [8]. Elite cues, such as those from public officials, can therefore play a prominent role in laying the groundwork for attitudinal shifts on the part of the public [9, 10]. For example, elite cues in the United States about climate change help drive media coverage and shape the attitudes of partisans, partially explaining why there are significant partisan differences in beliefs about the reality of climate change [11, 12]. Elites can also take cues from the public at times, but more often elites, through media access, influence public attitudes.

We evaluate the regulatory and policy landscape of AI adoption through a unique survey pool of 690 local officials in the United States—a representative sample of U.S. local officials. These are the kinds of officials that will make key decisions about AI adoption, including the regulation of these technologies and how government itself might adopt and implement them, given the decentralized structure of the United States. Thus, understanding this group is critical for assessing the way AI adoption will progress in the United States.

What motivates these local officials when they consider the potential utility of AI across different applications? The results are consistent with research on knowledge, familiarity, and technology adoption. Self-reported experience with AI is strongly correlated with increases in the likelihood of approval of AI uses, whether for autonomous vehicles and surgery, or other AI applications such as facial recognition, natural disaster impact planning, and military surveillance. Related, those who express opposition to AI adoption, particularly in controversial areas, are explicitly concerned with trade-offs between privacy and information and bias in algorithms.

Zeroing in on some of the most prominent potential uses of AI, local officials also appear significantly more supportive of autonomous vehicles than autonomous surgery, but conversely, worry about the safety issues arising from autonomous vehicles more (For prior work linking these two areas, see [13]). This is possibly due to a higher awareness of autonomous vehicles, given that regulatory decisions could be looming sooner.

Moreover, for autonomous vehicles, local officials differentiate between regulatory attitudes concerning development and adoption writ-large, and their personal willingness to use the technology. We demonstrate that a gap exists, and public policy support for autonomous vehicles is much higher than the individual willingness of respondents to use them.

Finally, we conduct a quantitative text analysis of the explanatory logic used by respondents when thinking about autonomous vehicles and surgery, and attitudes about algorithmic bias in general. The findings illustrate that how local officials think about different applications of AI depends on their prior experience with AI and on gender. For example, those with high levels of self-reported AI experience are more likely to rely on political, regulatory, and legal issues when making judgments about autonomous vehicles, and they are less likely to invoke personal experience when explaining their views about autonomous surgery. Meanwhile, women are more likely than men to use logic based on human control and values when making determinations about autonomous surgery, while women are less likely to describe their attitudes about autonomous vehicles as driven by their personal experiences.

In what follows, we outline some of the existing literature on attitudes surrounding AI adoption, discuss our survey sample and methods, describe the results, and then conclude.

## Literature review

The existing literature on attitudes about AI adoption includes both surveys of the general public in the United States and elsewhere, and the AI expert community, but little is known about the preferences of policymakers and those who will be implementing and regulating AI [14–22]. Those works centered on expert communities ask more granular and technical questions, such as what private and government actors are more trusted to manage and develop AI, how concerned are they about the ethical consequences of their work, and when do they think high-level machine intelligence (HLMI) will be realized [14, 23]. Broadening to the general public, the Partnership on AI’s Human-AI Collaboration Trust Literature Review surveyed 78 research articles on AI, humans, and trust. They found that, overall, while there was a general consensus that the trust humans place in AI depends on context, the literature appears to simplistically assume that explanations of the technology “will demonstrate trustworthiness, and once understood to be deserving of trust, people will use AI” [24]. This is consistent with work on the trust and adoption of algorithms [25, 26].

The current evidence about public and expert opinions about AI shows that attitudes vary across types of application and demographic factors. What can we learn from these to understand how local officials will make judgments about AI?

A Boston Consulting Group (BCG) survey of 14,000 internet users from around the world showed that respondents were relatively more supportive of AI uses in areas such as transportation and traffic, public infrastructure, and customer service [27]. Opposition was highest for AI use in contexts related to criminal justice. More generally, survey evidence from Europe suggests opposition to robotics is growing for robotics conducting work-related tasks traditionally accomplished by humans [28].

The most explored area surrounding attitudes about AI adoption is autonomous vehicles. Studies from around the world illustrate support, but also concern, about more vehicle automation. For example, similar to the desire for more transparency in attitudes about AI as a whole, surveys of the Australian public highlight that addressing questions of trust and safety are critical to public acceptance of autonomous vehicles [29]. A university student sample by [30] demonstrates that perceptions of security, privacy, and reliability inform trust in autonomous vehicles, and that trust and expectations about performance, in combination, drive attitudes about adoption.

One study of 5,000 respondents across the world found that concern surrounding autonomous vehicles was related to the potential for software hacking and misuse, in addition to legal issues [31]. Another study of respondents in the United Kingdom, United States, and Australia came to a similar conclusion [32].

More evidence about attitudes concerning autonomous vehicles comes from [33], who used the *Moral Machine* dilemma game to evaluate almost 40 million “decisions” about how users from 233 different countries, dependencies, or territories preferred an autonomous vehicle to handle a potential accident situation. The experiment revealed that while there were three dominant preferences—for prioritizing saving people, larger numbers of people, and younger people—that the differences varied across collectivistic and individualistic cultures. There is also evidence supporting variation in the acceptance of algorithms across cultures in general [34–37].

Healthcare applications for AI include the potential for algorithms to assist with planning a surgery, or even conduct surgery in some cases [38]. Most plans for autonomous surgery envision a human in at least a part of the loop—supervising the surgical procedure—though this would still be a substantial change from human-operated surgery [39]. Social media and content analysis have suggested there is growing optimism about increasing roles for AI in healthcare more broadly [40, 41]. For example, a survey of Korean doctors shows positive general attitudes about AI, but concern about the inherent limits of AI when faced with uncertain medical situations, particularly those with limited data [42]. Similarly, a September 2019 survey of patients in the United Kingdom finds support for algorithmic assistance with surgery, but opposition to the idea of fully autonomous surgery [43]. The BCG survey discussed above also highlights that respondents, on average, are comfortable with the use of AI to support medical diagnoses and recommendations for treatment, though the survey did not ask about treatment itself [27].

These surveys also highlight key demographic and individual factors that influence support for AI. As in research on technology adoption in general, the BCG survey shows that younger respondents and respondents that lived in urban areas were more supportive of AI than their older or more rural counterparts [27]. A study in Trikala, Greece found that younger people and those more accepting of automation were more likely to support autonomous buses [44]. One survey of the U.K. public illustrated that males and younger adults are more supportive of autonomous vehicles [45]. Like surveys in other countries, U.S. surveys found that younger and more educated respondents were more supportive of autonomous vehicles, though, overall, respondents were slightly more concerned than enthusiastic [16].

Familiarity with and knowledge of the technology also makes support and use of AI applications more likely. In [16], only 44% of respondents said they would ride in an autonomous vehicle. However, this number jumped to 57% when looking at the population that had “heard a lot” about the development of autonomous vehicles, compared to the 38% and 23%, respectively, of people who had “heard a little” or “not heard anything” prior to the survey [16]. Similarly, a survey of the public in Austin, Texas, found that individuals who had either been in a vehicular accident or had heard of Google’s self-driving car, were more likely to adopt autonomous vehicles earlier, and with less dependence on whether a significant portion of their friends had already adopted the technology [46]. Concern about bias and fairness also influences support [47, 48].

Given the existing literature, we would expect women and older respondents to be less supportive of AI adoption. Conversely, we would expect respondents with higher levels of education, and greater self-reported experience with AI to be more supportive of AI adoption.

## Materials and methods

In federal countries like the United States, local officials will play a huge role in determining AI adoption. Regulation in the United States is often a patchwork of local, state, and national-level regulation. There is also not a lot of data and analysis comparing different localities and states on public policy issues [49].

To address the gap in knowledge about U.S. local attitudes, we draw on a survey of 690 local officials in the United States fielded by CivicPulse in October 2020 [49]. The study was ruled exempt by the IRB at the University of Pennsylvania, protocol 828933. Participants were all public officials, so they are exempt and non-vulnerable. They were guaranteed confidentiality by CivicPulse. 555 respondents completed the full survey, while 135 completed part of the survey. We use probability weights that mirror the strategy used for the American National Election Survey to ensure the representativeness of the results for local officials in the United States [50]. However, as S4–S6 Tables in S1 File show, the results are consistent whether or not we use probability weights.

**Fig 1:**
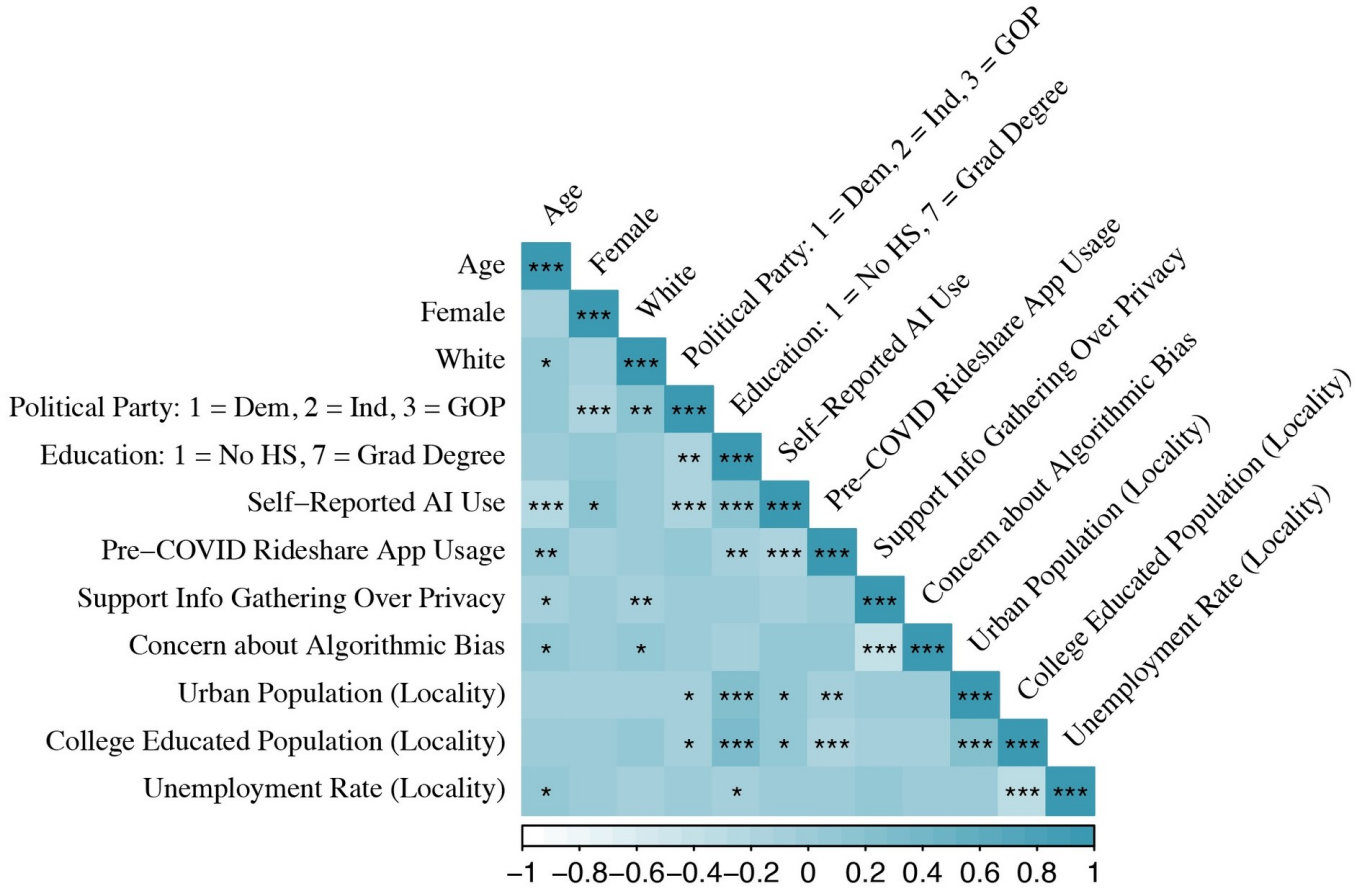
Demographic correlation matrix.

Fig 1 below shows a correlation matrix between key demographic attributes of survey respondents. The correlations are in expected directions, ex-ante. For example, districts with higher percentages of urban footprints are also more likely to have more college graduates, while female respondents are more likely to be Democrats. Older respondents are less likely to self-report using AI at home or work, or in how they consume music and movies. Republicans also appear less likely to self-report using AI. The results also suggest that those respondents that reported greater levels of concern about algorithmic bias are more likely to prioritize individual privacy over information gathering by the government.

The sample is comprised of local officials in the United States and it skews older than the average U.S. adult population. The average respondent is between 55–60 years old, as opposed to 38 years old for the U.S. adult population as of 2018 [51].

## Results

The results demonstrate the evolving attitudes of local officials in the United State surrounding AI-enabled systems, especially autonomous vehicles and autonomous surgery, in addition to broader uses of AI. Below, we highlight how demographic factors, knowledge and interest in issues surrounding artificial intelligence, and other variables influence these attitudes. We do this via regression analysis, first for autonomous surgery and autonomous vehicles more specifically, and then for other applications of AI. We also conduct text analysis to draw out sentiments and explanations for why certain factors might influence support for autonomous surgery and autonomous vehicles, and we also focus more narrowly on the gaps between levels of stated support versus willingness to use for autonomous vehicles.

### Autonomous vehicles and autonomous surgery

**Fig 2:**
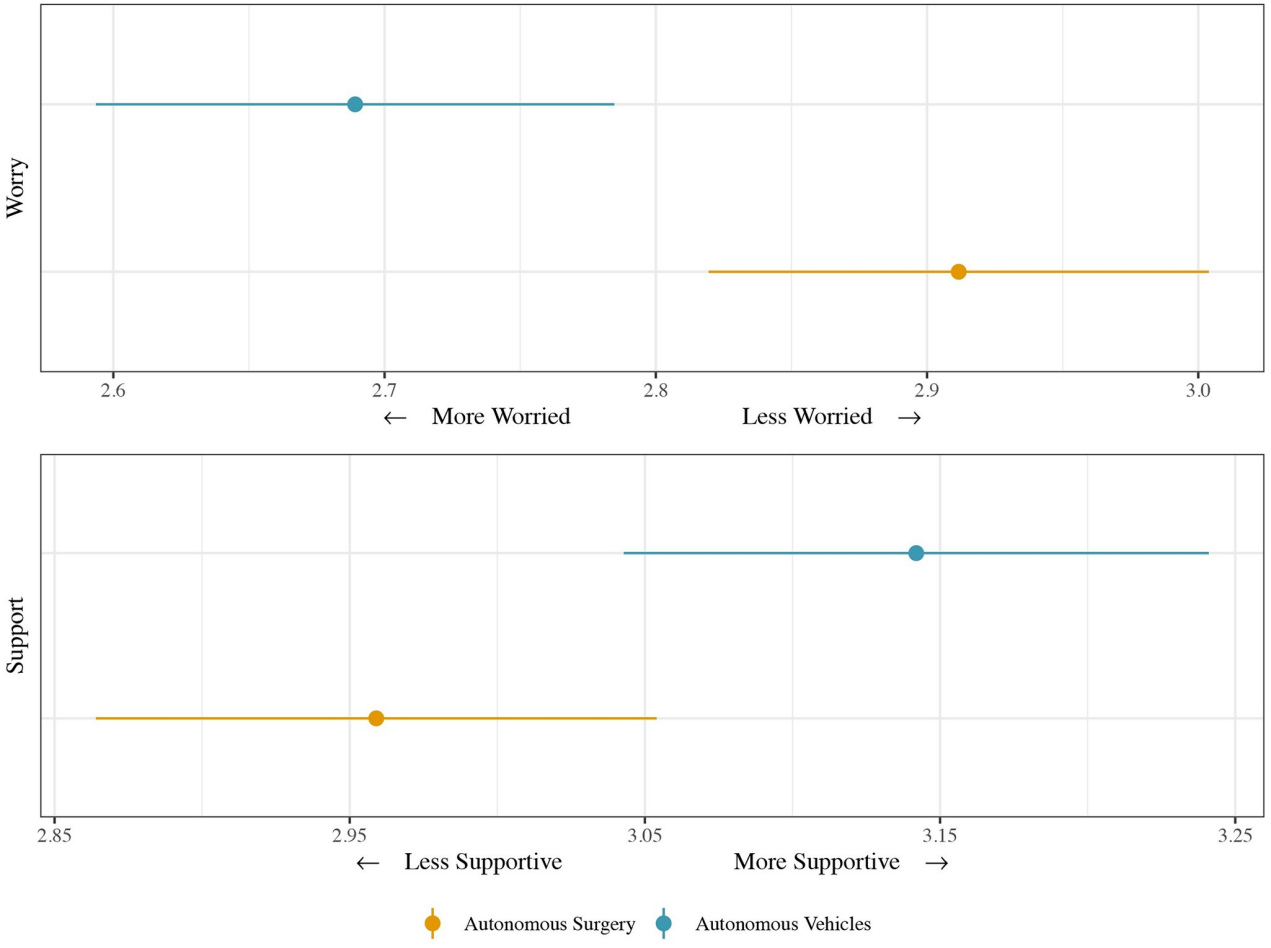
Mean levels of support and worry for autonomous vehicles and surgery.

We start by showing the average response values of local officials to questions about their policy support for autonomous vehicles and autonomous surgery, including their concerns about safety issues surrounding these applications. Overall, as Fig 2 shows, there is more support for autonomous vehicles, pooled across states and localities, than for autonomous surgery. Respondents answered a question about public policy support for autonomous vehicles and surgery on a 1–5 Likert scale, where 1 represented “strongly support” and 5 “strongly oppose.” The average level of public policy support for autonomous vehicles is 3.14, suggesting attitudes are relatively positive, while the average level of public policy support for autonomous surgery is 2.96, just below the response midpoint.

As a follow-up, respondents were asked about their concerns about the safety of autonomous vehicles and autonomous surgery on a 5-point Likert scale, where 1 reflected “extremely worried” and 5 “not worried at all.” As Fig 2 shows, for both potential AI use areas, the mean responses were below 3, meaning respondents were, on average, worried. Respondents were also relatively more worried about potential safety issues arising from autonomous vehicles than from surgery, despite expressing more support for autonomous vehicles. This result would exist because, in accident situations, more humans are likely to be harmed by an autonomous vehicle failure than an autonomous surgery failure. Surgery is inherently dangerous, with only the patient at risk. With autonomous vehicles, a car crash can harm both those within and outside the vehicle, such as pedestrians, cyclists, or other drivers or passengers. These results are therefore consistent with the *Moral Machine* experiment [33].

#### Regression models

What explains these differences? To explore the possibilities, we estimate a series of regression models designed to test how individual differences and demographics of states and localities influence support for autonomous vehicles and autonomous surgery. The dependent variables for the regression models are support for the use of autonomous vehicles and support for the use of autonomous surgery, respectively, using the same Likert scale variables described above. We use ordinary least squares regression for these models given the 1–5 coding of the dependent variables. We show in the S7–S9 Tables in S1 File that these results are robust to an ordinal logit estimation strategy. We cluster standard errors by state given the possibility for unmeasured variation across U.S. states, and weight observations to make the sample representative of the population of local officials. We show in the S4–S6 Tables in S1 File that the unweighted results are substantively identical.

We include the following independent variables in the regression models.

- Sex
- Age
- Level of education
- Political party
- Use of ridesharing apps (pre-COVID)
- Does respondent live in top 10 auto manufacturing state (Vehicles model only). Data from [52].
- Percentage of employment from hospitals (Surgery model only). Data from [53].
- Self-reported AI use
- Relative support for information gathering versus privacy protection
- Level of concern about algorithmic bias

Details on the measurement of all items are available in the S3 File. We also include variables to control for the economic and political environment facing local officials, including the percentage of the population that lives in an urban setting, the percent that is college-educated, and the unemployment rate.

**Fig 3:**
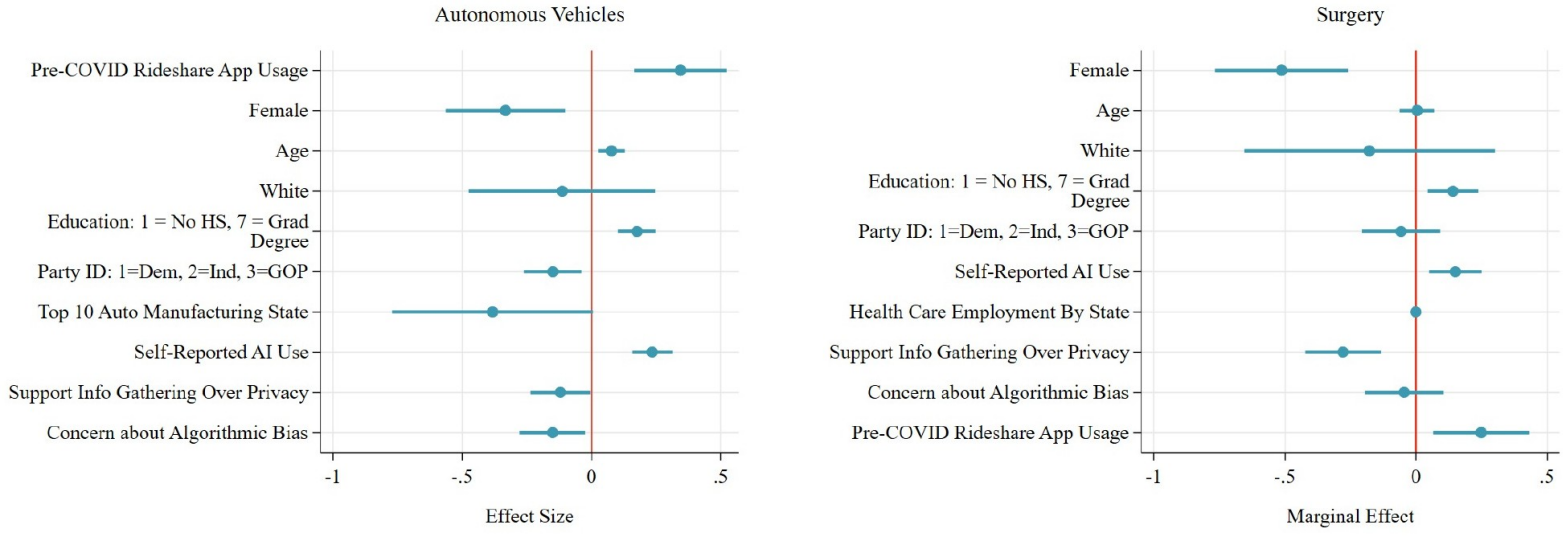
Drivers of attitudes about autonomous vehicles and surgery.

The results in Fig 3 highlight key regulatory and use attitudes of U.S. local officials concerning autonomous vehicles and surgery. Since the regression model is OLS, we can interpret the substantive effects of the coefficients directly. Consistent with prior research, women are significantly less supportive of autonomous vehicles and autonomous surgery than men. Republicans are also less supportive in both usage cases, though the effect is not statistically significant for autonomous surgery.

Higher levels of education are associated with more support for both autonomous vehicles and surgery. In contrast to prior literature, older respondents are more supportive of AI adoption as well. The uniqueness of the sample, which is older than the general public, may explain this result, in part. Officials who live in top 10 auto manufacturing states such as Michigan and Illinois are less likely to support policy adoption of autonomous vehicles, though there is no equivalent effect for autonomous surgery for those in top healthcare employment states.

Those that reported using ridesharing apps like Uber and Lyft frequently prior to the COVID-19 pandemic are 23.5% more supportive of adopting autonomous vehicles than those who did not use them frequently. Previous ridesharing app usage might lead to higher support for autonomous vehicles because using an autonomous vehicle requires delegating driving decisions away from oneself, and trusting a machine. Ridesharing app users have already made that delegation decision, albeit transferring control to another individual, rather than to an algorithm. This reduces the cognitive “leap” required to delegate driving to an AI. Those that have not used ridesharing apps both have to make the initial judgment to delegate and decide to delegate to an algorithm.

We measure self-reported experience with AI through two questions. One asked if respondents used AI at work, at home, both, or neither. Another question asked respondents if they use music or movie services (like Pandora or Netflix) where algorithms generate media recommendations. We aggregate the results to create an index that runs from 3 (greater self-reported AI use) to 0 (no self-reported AI use). The results show the strong, positive effect of self-reported AI use on support for the use of AI in other areas. A one-unit increase in self-reported AI use is associated with a 0.235 (*p* < 0.01) increase in support for autonomous vehicles, and a 0.165 (*p* < 0.01) increase in support for autonomous surgery. Those more concerned about the potential for bias in algorithms and those more concerned with privacy intrusions due to AI are less likely to support either autonomous vehicles or autonomous surgery.

#### Regulatory support versus personal use

Another question—specifically for autonomous vehicles—allows us to differentiate between respondent attitudes about public policy choices and their personal views. We asked respondents whether they would personally ride in a self-driving car. This allows us to measure the potential gap between expressed policy attitudes and personal beliefs about usage.

The results show, for the most part, higher levels of policy support for autonomous vehicles than a willingness to use them themselves. On an individual level, the gap between a respondent’s likelihood to support the development of autonomous vehicle technology and personal use can be plotted on a scale from -3 (where a respondent was more likely to use than to support) to 3 (where a respondent was more likely to support than to use). The mean gap was 0.452. While 51% of respondents had a gap of 0, suggesting consistency in their willingness to support and use autonomous vehicles, 39% had a positive gap, meaning their degree of policy support was higher than their willingness to use, only 10% suggested they would be more likely to use the technology themselves than support its development. Officials who said they would be more likely to use the technology themselves than support it are primarily from communities with a smaller percentage of college-educated adults. This is consistent with the results discussed above, since those with higher levels of education are generally more supportive of AI applications. It is possible that local officials in these areas with fewer college-educated adults predict lower levels of support amongst their constituents, and therefore do not advocate for broad adoption, even though they would be willing to use the technology themselves. Women were more likely to support autonomous vehicles more broadly than indicate a willingness to use. This is consistent with prior research that women are more cautious when it comes to new technology use, and more specifically autonomous vehicles and surgery [54].

**Fig 4:**
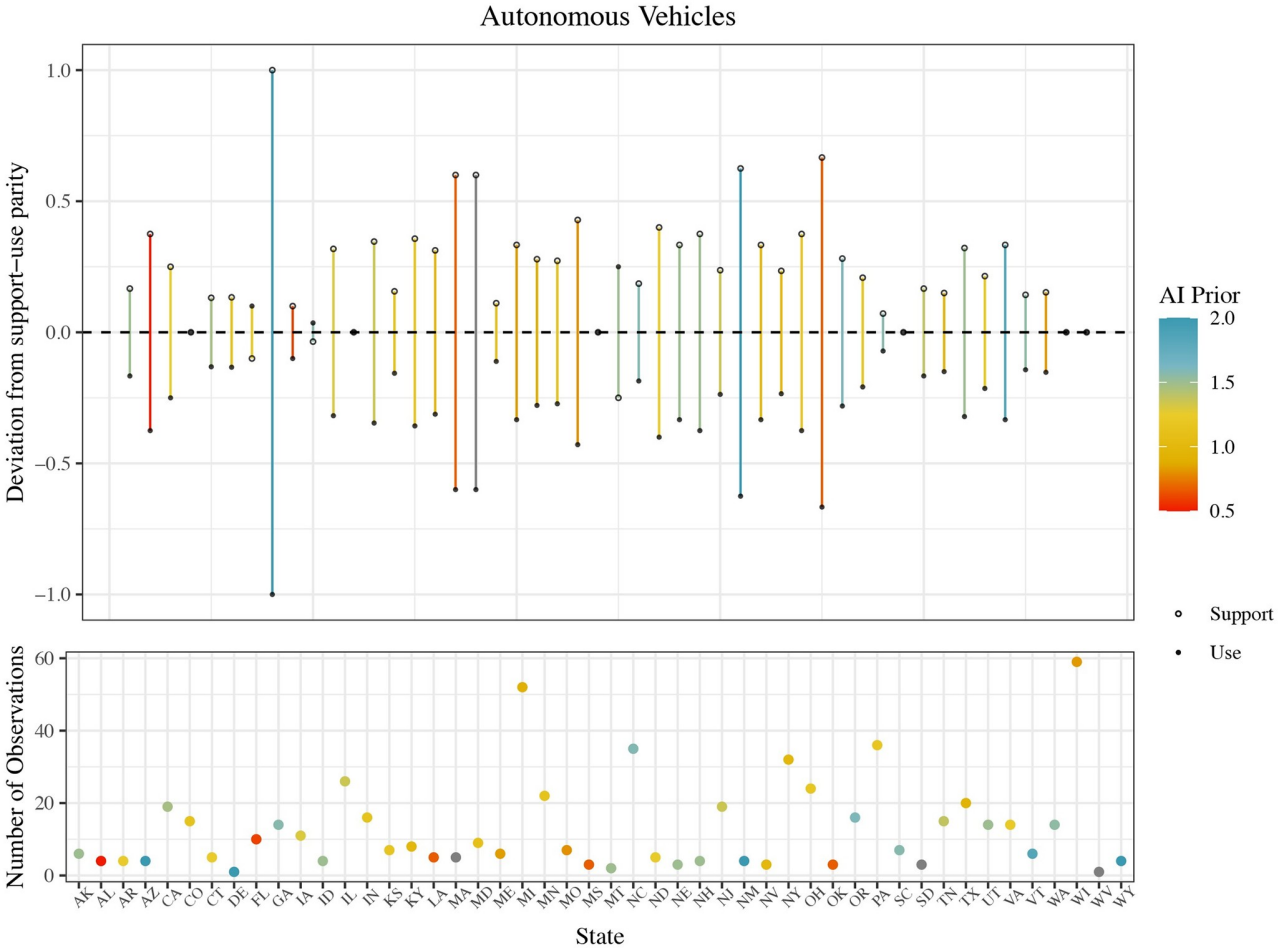
Gap between public policy support and personal use willingness, by state, for autonomous vehicles.

A similar trend is evident when we aggregate the individual-level data to the state level, where the average gap ranged from -0.5 to 2, with a mean of 0.471. Out of the 48 states represented for these questions, 9 had a gap of 0, 39 had an average level of support higher than a desire to use the technology, and only 3 states had a negative gap, meaning their personal usage average was higher than their policy support average. Fig 4 below illustrates the size of the average gap between policy support and willingness to use for each state, along with the number of observations for each state, and the average level of self-reported use of AI. Respondents in the top 10 auto industry states had an average gap size of 0.55, while those in non-top 10 auto industry states had an average gap size of 0.47 (*t*-tests verified the difference is statistically significant at the 0.05 level).

One interpretation of these results is that the personal willingness to use represents revealed preference about attitudes, meaning respondents’ answer to the question about public policy support over-estimates that support. A second interpretation is that local officials are attempting to be open-minded from a regulatory perspective about autonomous vehicles, even if they are personally concerned about whether the technology is ripe enough to use. Future research could untangle these differences.

#### Text analysis

To further explore the mechanisms underlying these results we analyze open-response questions from our survey instrument. These open-response questions asked respondents to explain their support or opposition to autonomous vehicles and surgery. Each open-response question was asked immediately after the relevant quantitative question about their support or opposition. The full survey text is available in S3 File. Two independent coders then evaluated the text responses. First, coders categorized the responses based on the logic used by the respondent. Second, coders judged whether the use of that logic was expressed positively, neutrally, or negatively with regard to AI. Further coding details are in S4 File. Coders could choose up to three categories for each response, assigning a positive/neutral/negative rating to each category. When the coders disagreed in their category assignments or sentiment, the authors arbitrated the disagreement and, when necessary, inserted a third coding to break the tie. The categories are as follows:

- Control
- Human Values
- Legal/Regulatory/Political
- More Information Needed
- Personal Experience
- Safety (Includes Machine and Human Safety)
- Societal Impact (Includes Implementation)
- Technical Reliability

**Fig 5:**
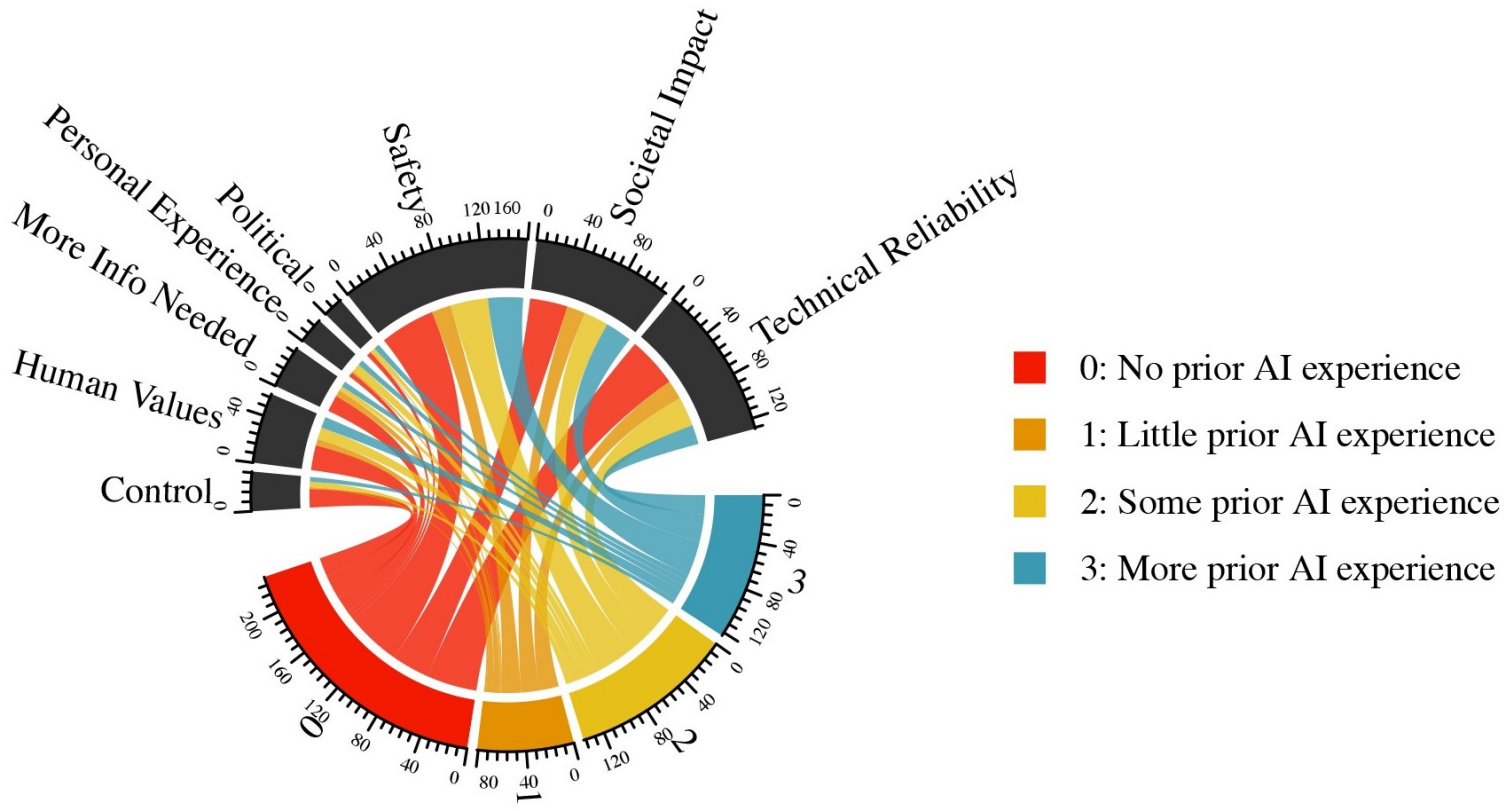
Respondent sentiments on autonomous vehicles, by level of prior experience with AI.

Fig 5 highlights how self-reported experience with AI makes respondents more or less likely to use different types of reasoning when explaining their views on autonomous vehicles. For example, respondents with no self-reported AI experience disproportionately cited control and lack of information as reasons for their support or lack thereof for autonomous vehicles. While only 40% of overall respondents had zero self-reported experience with AI, 60% of those citing *Control* and 50% of those citing *More Information Needed* had zero self-reported experience with AI. In contrast, those with the highest level of self-reported experience with AI were more likely to invoke political or regulatory explanations for their views on autonomous vehicles, and less likely to discuss technical reliability, control, or ask for more information.

**Fig 6:**
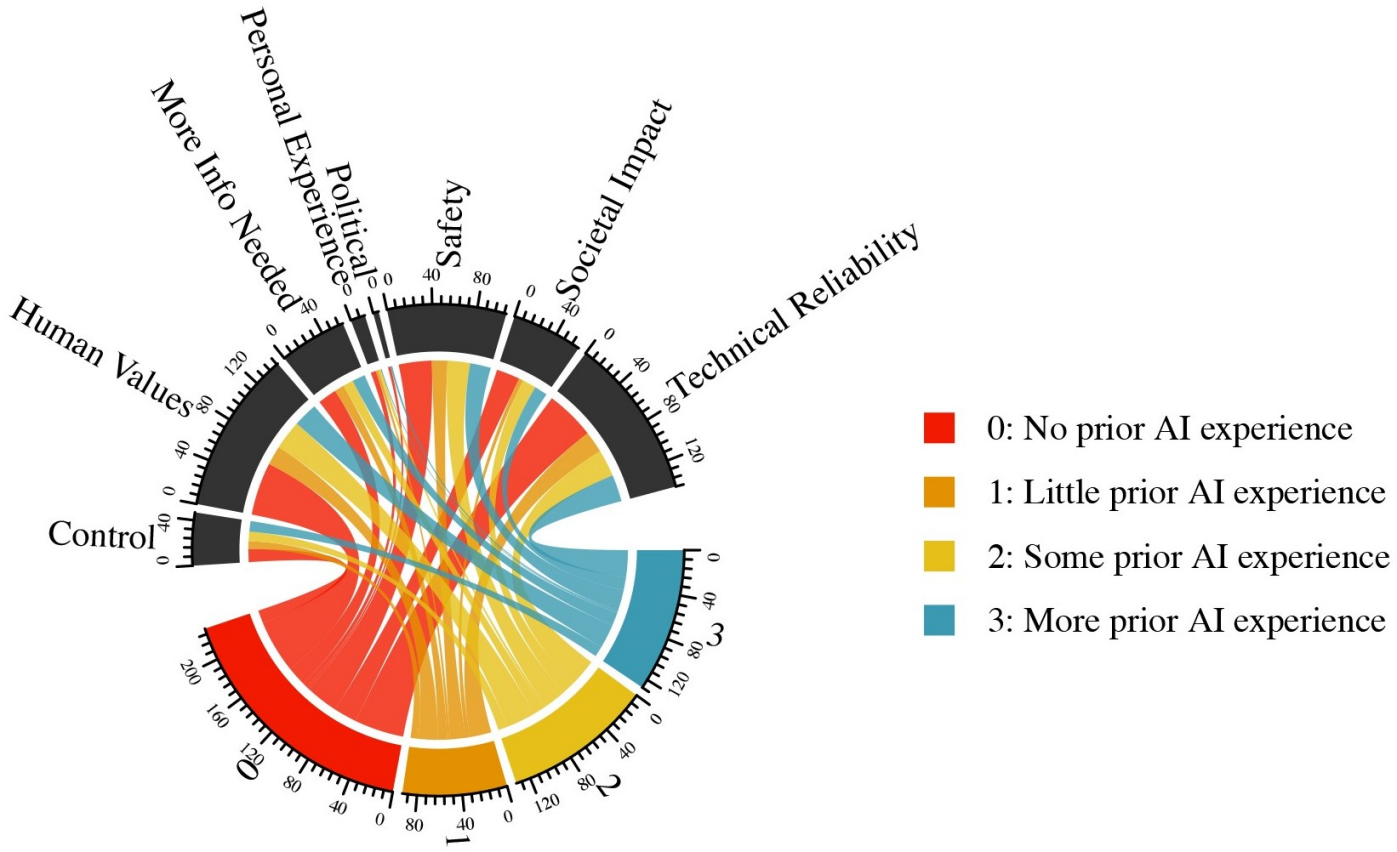
Respondent sentiments on autonomous surgery, by level of prior experience with AI.

The autonomous surgery text analysis results shown in Fig 6 differ from the autonomous vehicles results. In contrast with the autonomous vehicle results, those with no self-reported AI experience were less likely to use arguments about control when explaining their views about autonomous surgery. They were more likely, however, to make arguments based on personal experience. For those with higher degrees of self-reported AI experience, the explanatory logic they used largely aligned with their representation in the population of respondents, with one exception. Those with higher levels of self-reported AI experience were significantly less likely to use logic based on personal experience. For example, while 22% of respondents to the autonomous surgery question reported high levels of self-reported AI experience, only 7% of the respondents who invoked personal experience came from that subgroup. One possible explanation for these results could be that those with higher levels of self-reported AI experience were more likely to understand how AI might be implemented in healthcare, and therefore less likely to rely on personal experience or heuristics to explain their responses.

Overall, when comparing reasoning for support for autonomous vehicles with reasoning for support for autonomous surgery, *Safety*, *Societal Impact*, and *Technical Reliability* were the three most cited for vehicles, while *Human Values*, *Technical Reliability*, and *Safety* were the most frequent logics for surgery.

**Fig 7:**
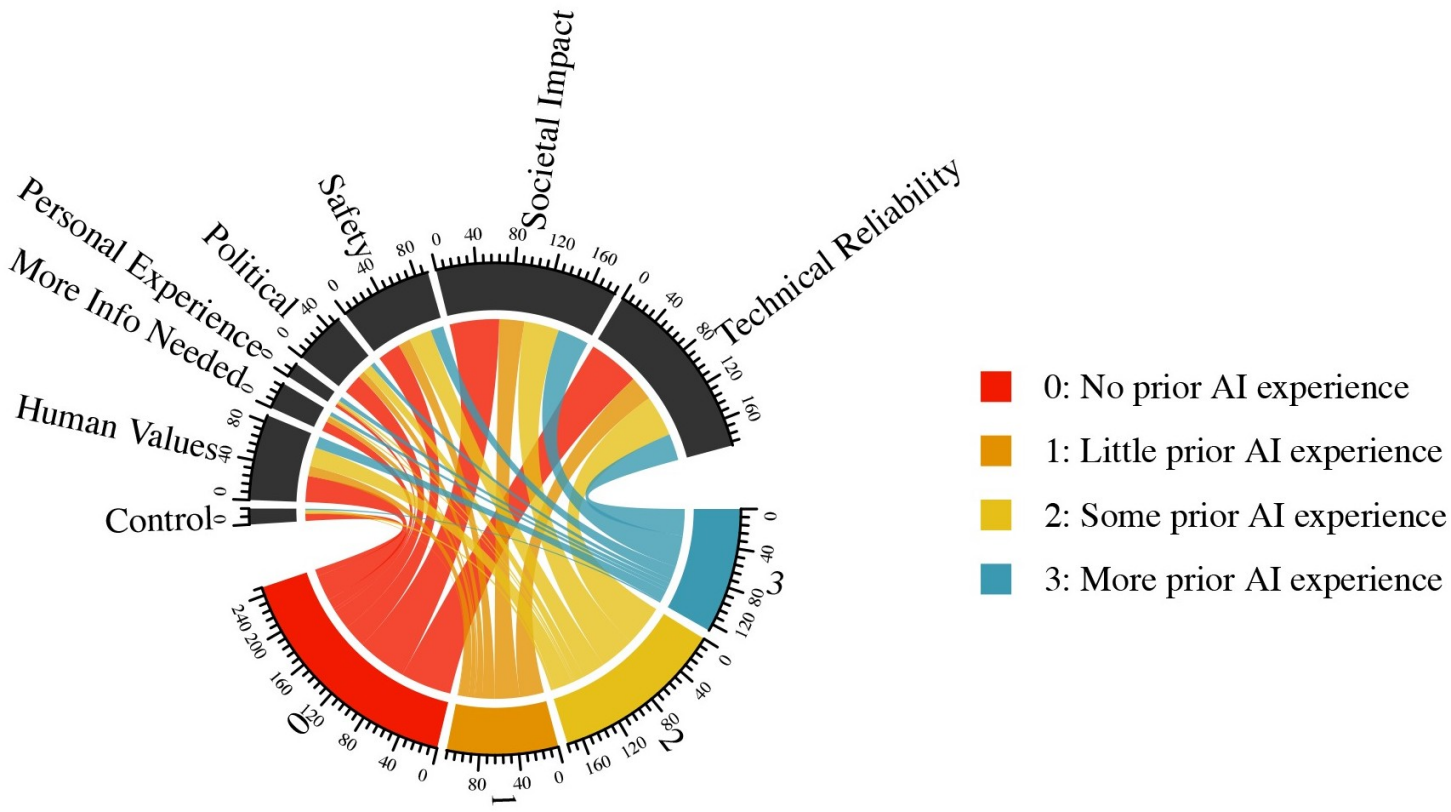
Respondent sentiments on bias in algorithms, by level of prior experience with AI.

Finally, we evaluate respondent explanations about their level of concern about AI bias, further unpacking a key independent variable in the analyses described above. These results are in Fig 7. Those with no self-reported experience with AI were relatively less likely to use safety or personal experience explanations in identifying their reasoning, but more likely to use the logic of maintaining human control. Alternatively, those with relatively higher levels of self-reported AI experience explained their views on AI bias much more through referring to their personal experience, and much less by discussing control or raising issues surrounding politics or regulatory questions.

**Fig 8:**
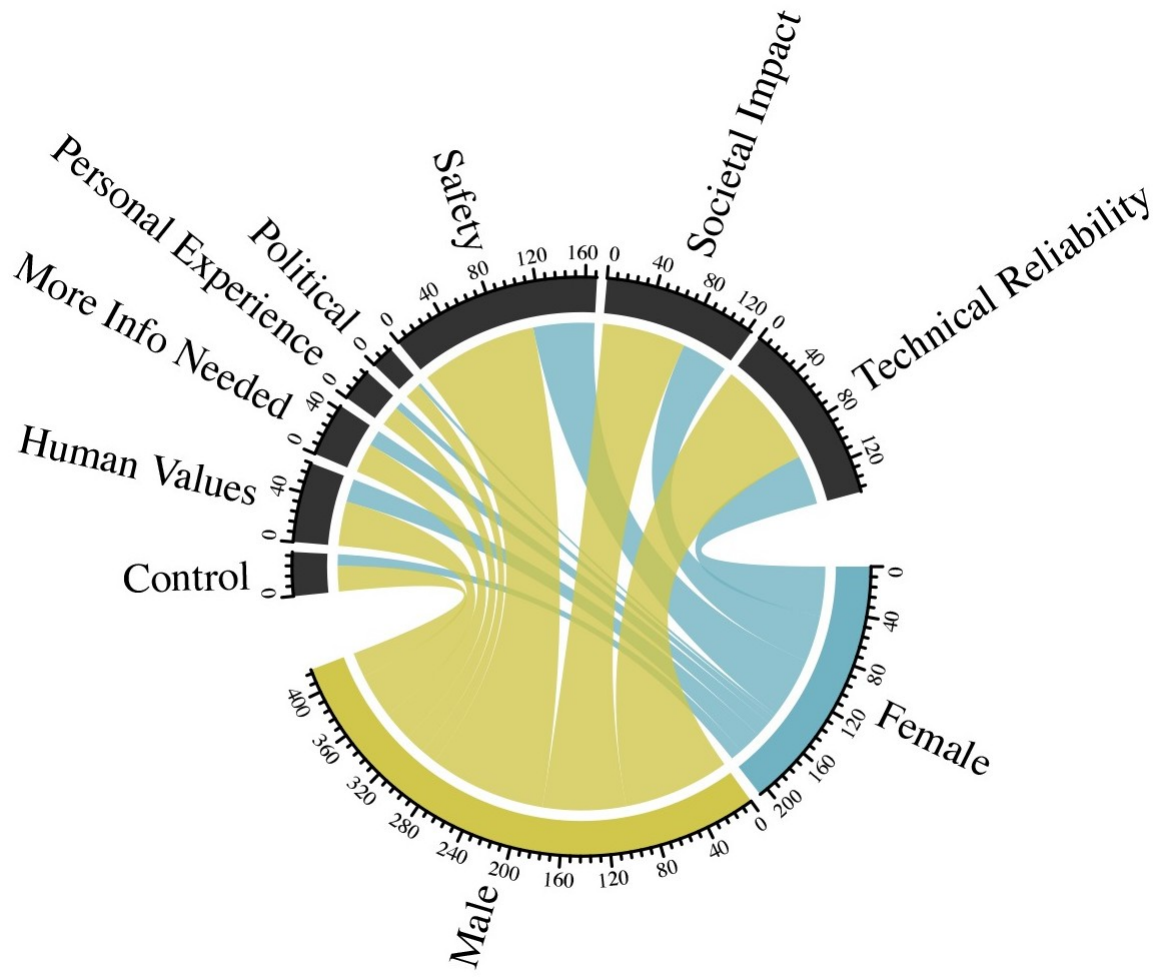
Respondent sentiments on autonomous vehicles, by gender.

**Fig 9:**
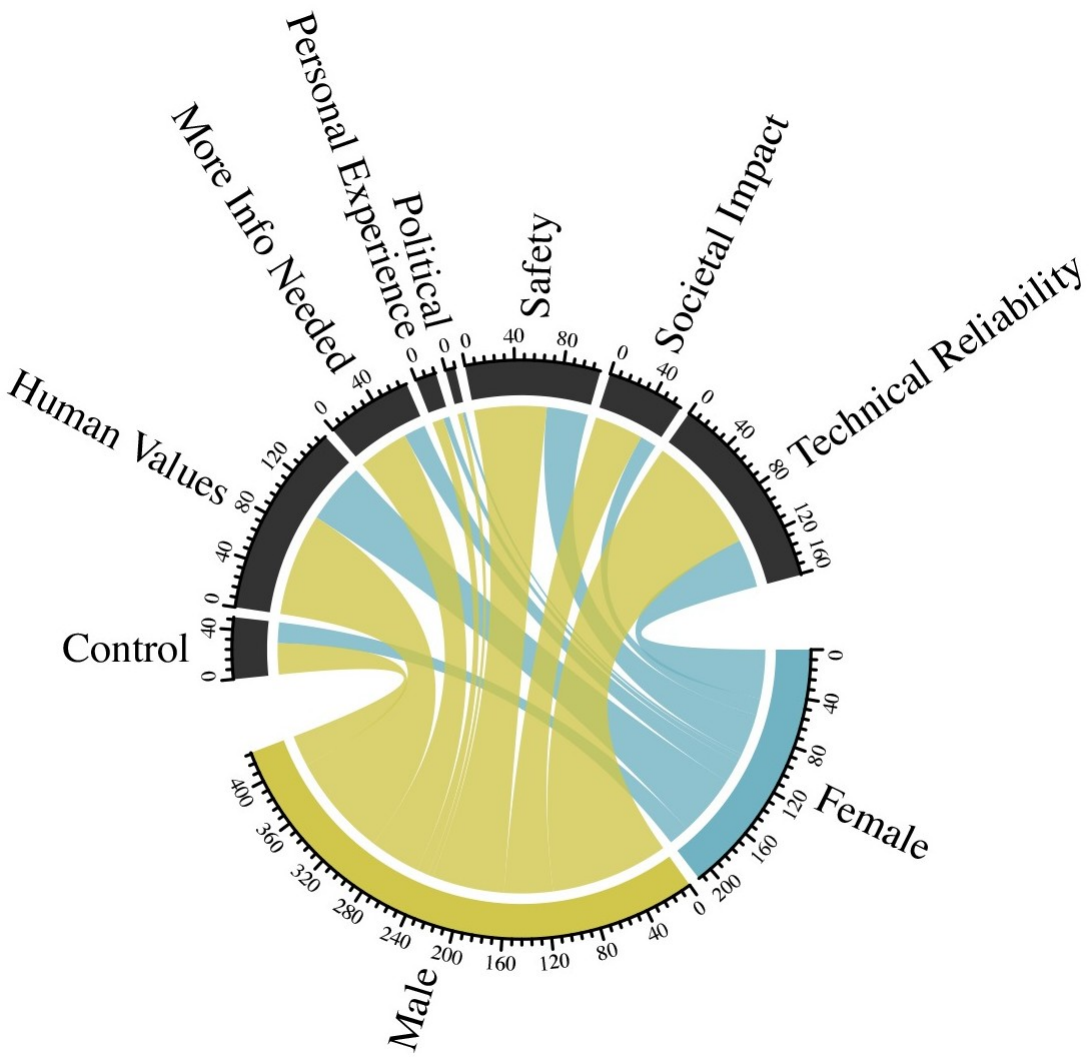
Respondent sentiments on autonomous surgery, by gender.

Figs 8 and 9 further show how gender influences the explanatory logic used by respondents when thinking about autonomous vehicles and surgery. As described above, our regression models illustrate that women were less likely to support autonomous vehicles and surgery than men. Using text analysis, we can compare how frequently women and men used different types of reasoning when describing their attitudes about autonomous vehicles and surgery, in comparison with what we would expect based on their prevalence in the sample as a whole. S5 and S6 Figs in S1 File show sentiment analysis from these open response questions that match the relatively higher levels of skepticism expressed by women about autonomous vehicles and surgery.

Women were substantially less likely to describe their attitudes about autonomous vehicles as driven by their personal experience or political/regulatory questions surrounding autonomous vehicles. For example, women represent 33% of the overall sample, but only 17% of those that emphasize legal, regulatory, and political arguments. Women were slightly more likely to use explanations focused on societal and implementation issues associated with AI. When it comes to attitudes about autonomous surgery, women were less likely to make arguments about societal consequences, and more likely to make claims based on control.

### Other uses of artificial intelligence

We now turn from attitudes about autonomous vehicles and surgery to the beliefs of local officials about other uses of AI. We asked respondents to give their opinions on each potential use of AI on a Likert scale from “very unsupportive” to “very supportive”, with “no opinion” also a response option. The other uses of AI were:

- Surveillance of criminal suspects through facial recognition software and other means
- General monitoring of the civilian population for illicit or illegal behavior
- Job selection and promotion for local officials
- Decisions about prison sentences
- Decisions about the transplant list
- Natural disaster impact planning
- Responding to 911 calls
- Surveillance and monitoring of military targets
- Use of military force

**Fig 10:**
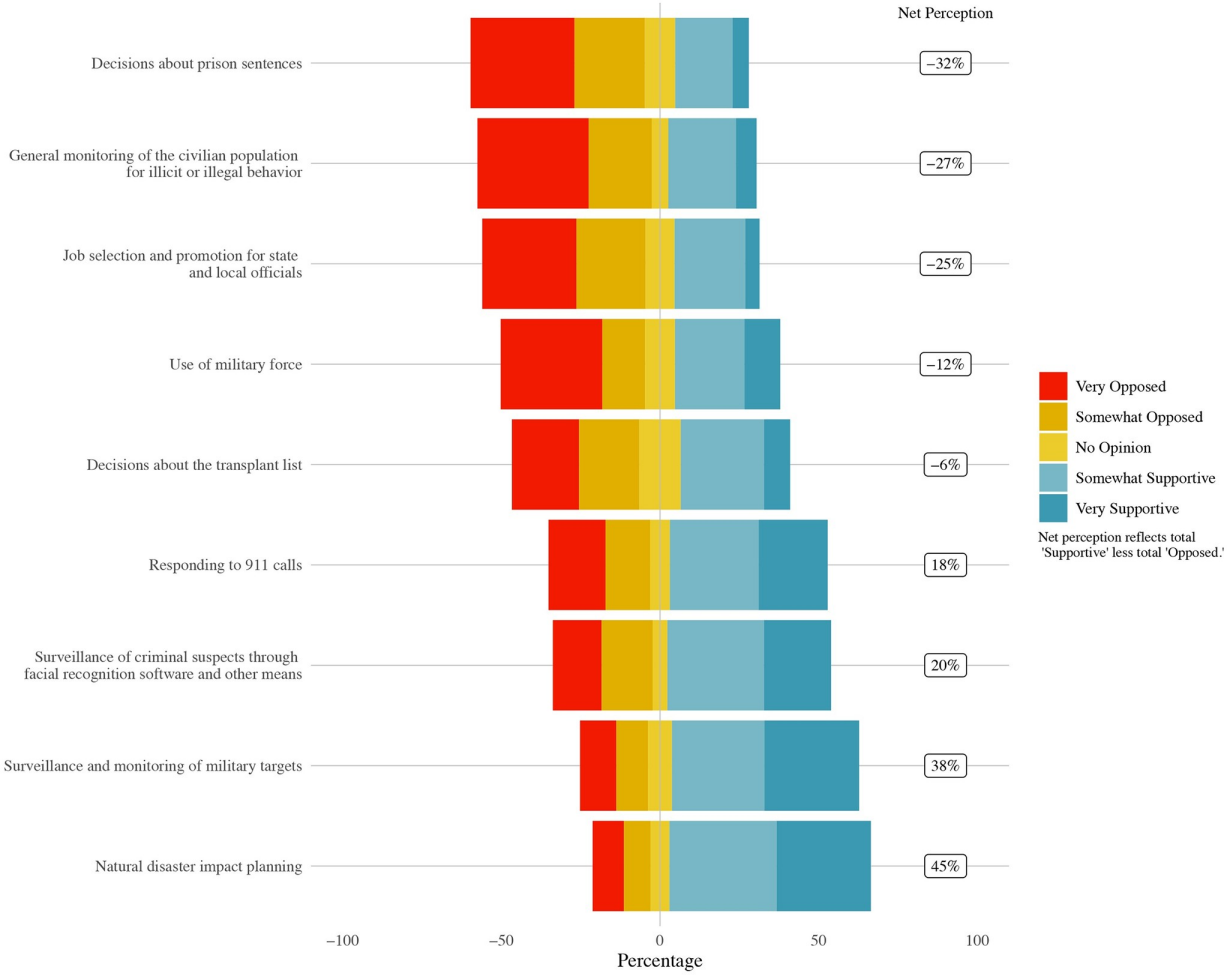
Levels of support for AI adoption for local officials.

These items range from applications thought to be relatively uncontroversial, like natural disaster impact planning, to controversial regulatory areas for local officials, like criminal surveillance and population monitoring, to broader potential uses of general interest, such as military applications. Fig 10 below shows the distribution of attitudes across these questions.

The results illustrate widely varying attitudes across these AI use cases. Uses of AI with the largest net support levels include natural disaster impact planning (45%), surveillance and monitoring of military targets (38%), surveillance of criminal suspects (20%), and responding to 911 calls (18%). Local officials had net negative perceptions of all other AI applications, especially decisions about prison sentences (-32%), and general monitoring of the civilian population (-27%). Perhaps not surprisingly from a bureaucratic politics perspective [55], local officials had a net -25% view of using AI for job selection and promotion for local officials.

As in surveys of the general population and AI experts, such as [21, 23], there is a gap between support for military surveillance uses of AI and support for AI being employed in the use of military force. Local officials had a -12% perception of using AI for military force—a 50% differential from the +38% perception of military surveillance. This gap highlights the importance of distinguishing between types of military uses of AI. Local officials in the United States appear to share some of the same hesitations about the use of AI for the use of force also found in AI expert surveys and surveys of the general public [56].

#### Regression models

To further explain the drivers of support and opposition for AI uses, we turn to the same OLS regression strategy used above to illustrate the drivers of support for autonomous vehicles and autonomous surgery. Table 1 displays the results, which show key similarities with the findings for autonomous vehicles and autonomous surgery.

**Table 1:** Drivers of attitudes about uses of AI.

	(1) Facial Recognition Software	(2) General Monitoring	(3) State Jobs	(4) Prison Sentences	(5) Transplant List	(6) Natural Disaster Impact Planning	(7) Responding To 911 Calls	(8) Military Surveillance	(9) Use of Military Force
Female	✘	✘	✘	✘	✘	✔	✔	✘	✔
Age	✔	✘	✘	✔	✔	✘	✔	✔	✘
White	✘	✘	✘	✘	✘	✘	✘	✘	✘
Education: 1 = No HS, 7 = Grad Degree	✘	✘	✘	✔	✘	✘	✘	✘	✘
Party ID: 1 = Dem, 2 = Ind, 3 = GOP	✔	✘	✘	✘	✔	✘	✔	✘	✔
Pre-COVID Rideshare App Usage	✘	✘	✘	✘	✘	✘	✘	✘	✘
Self-Reported AI Use	✘	✘	✔	✘	✔	✔	✔	✔	✘
Support Info Gathering Over Privacy	✔	✔	✔	✔	✔	✘	✔	✔	✔
Concern about Algorithmic Bias	✔	✔	✔	✔	✔	✔	✔	✔	✔
Urban Population	✘	✘	✘	✘	✘	✘	✘	✘	✘
College Educated Population	✘	✘	✘	✘	✔	✔	✘	✘	✘
Unemployment Rate	✘	✘	✘	✘	✔	✘	✘	✘	✘
Observations	445	438	419	420	407	439	437	430	419

Checkmark denotes significant at the *p* < 0.10 level or above. The full regression table is in S1 Table in S1 File.

The most consistently significant driver of support for these AI applications is self-reported experience with AI. Moreover, those concerned with algorithmic bias and those that view protecting privacy as more important than gathering information also were significantly less likely to approve of uses of AI in most areas. Natural disaster impact planning is the only case where the information versus privacy dilemma does not predict respondent support for an application of AI. For those potential AI uses that were the least popular among local officials, such as general monitoring of the population and local job selection and promotion, the only significant predictors of respondent attitudes were these variables measuring broader attitudes about and experience with AI.

The impact of gender is noticeable—women were less likely to support the use of AI in some, but not all, of the AI use cases. Women were less likely to support the use of AI even in less controversial cases such as natural disaster impact planning. Partisanship also helps us understand many of these responses. Republicans and Republican-leaning independents were more likely to support the use of AI for facial recognition surveillance of criminal suspects and the use of military force, while Democrats and Democrat-learning independents were significantly more likely to support the use of AI for transplant list decisions and responding to 911 calls.

## Limitations

While these results shed new light on attitudes surrounding AI adoption in multiple arenas, this study has limitations as well. The sample is only representative of local officials in the United States. Follow-up research could focus on U.S. federal officials (both inside and outside the national security domain), state officials, and similar local officials in other countries. Otherwise, it is hard to know to what extent these results might generalize outside local government in the United States.

One might also have concerns about non-response bias, given that the number of responses to the second block of questions about AI approval dropped off by 77. We randomized the order in which respondents saw the autonomous vehicles block of questions, the autonomous surgery block of questions, and the broader AI use applications block of questions, which mitigates this concern to some extent. However, to ensure non-responses did not bias the results, we reduced the sample down to just the respondents who completed all nine questions in S1 Table in S1 File along with the other questions and re-ran the models applied to autonomous vehicles and autonomous surgery. As S3 Table in S1 File shows, the results are substantively identical to our main results, increasing our confidence in the overall results.

## Conclusion

How countries, states, and local actors make choices about the adoption and use of AI will play a vital role in shaping how AI impacts societies around the world. Specifically, local actors, especially in federal countries like the United States, will make key legal and regulatory decisions surrounding the use of AI in areas such as transportation, healthcare, and policing, just as they make policy decisions about those areas in general. In this paper, we take advantage of a novel sample of local US officials from the CivicPulse project to explore the attitudes of this crucial population surrounding artificial intelligence.

The results show higher levels of support for autonomous vehicles than autonomous surgery, and a substantial gap between policy and regulatory attitudes surrounding autonomous vehicle adoption on the one hand, and personal willingness to use autonomous vehicles on the other hand. Policy support is much higher than willingness to use. Moreover, respondents had more safety concerns about autonomous vehicles than autonomous surgery, despite favoring autonomous vehicles more.

Self-reported experience with artificial intelligence plays a prominent role in shaping attitudes about adoption. For autonomous vehicles and autonomous surgery, as well as a battery of additional AI use cases, those that describe themselves as already using AI were consistently more supportive. This aligns with existing literature that suggests, generally speaking, understanding of technology leads to higher levels of trust.

Finally, these factors also shape the types of reasoning and logic they use when explaining their views. Our text analysis demonstrates that both prior experience with AI and gender significantly shape the way people think about AI. Overall, these results shed new light on the role of AI in the United States, and how policy and regulatory decisions might evolve, with the technology, in the years to come.

## Supporting information

S1 File Additional tables and figures. (PDF)

S2 File Variable definitions. (PDF)

S3 File Survey text. (PDF)

S4 File Text analysis coding scheme. (PDF)
