# Supplementary material for: What influences attitudes about artificial intelligence adoption: Evidence from U.S. local officials
Source: PLoS One. 2021 Oct 20;16(10):e0257732. doi: 10.1371/journal.pone.0257732 (PMC8528275; doi:10.1371/journal.pone.0257732)
Supplement: S1 File — (PDF) [file pone.0257732.s001.pdf]

S1 Additional Tables and Figures.

Data and replication code available at: https://doi.org/10.7910/DVN/BUYRQG.

S1 Fig. Levels of Support for AI Adoption for Local Officials: Smaller Sample.

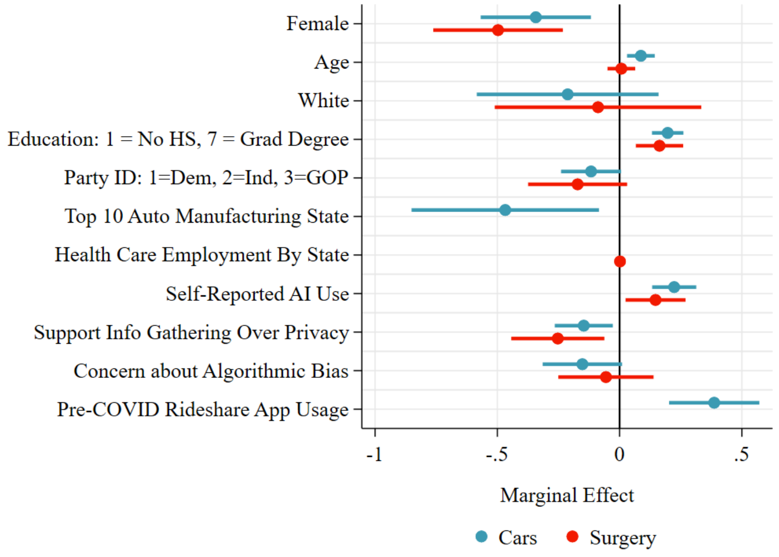

S2 Fig. Respondent sentiments on autonomous vehicles, by level of prior experience with AI.

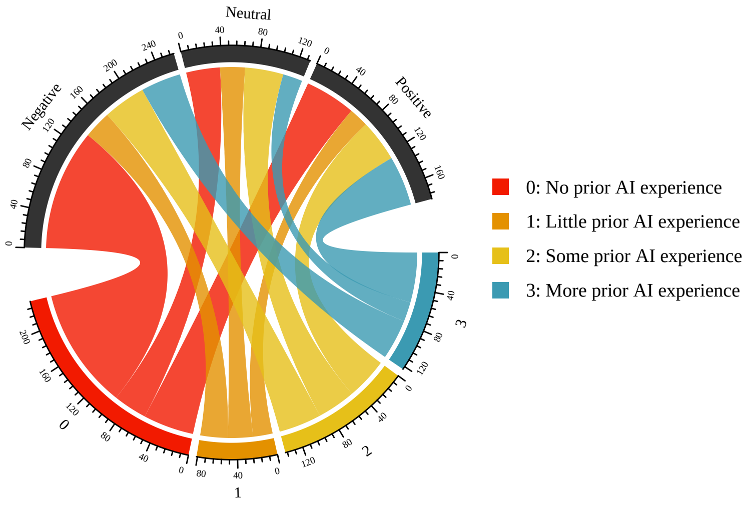

S3 Fig. Respondent sentiments on autonomous surgery, by level of prior experience with AI.

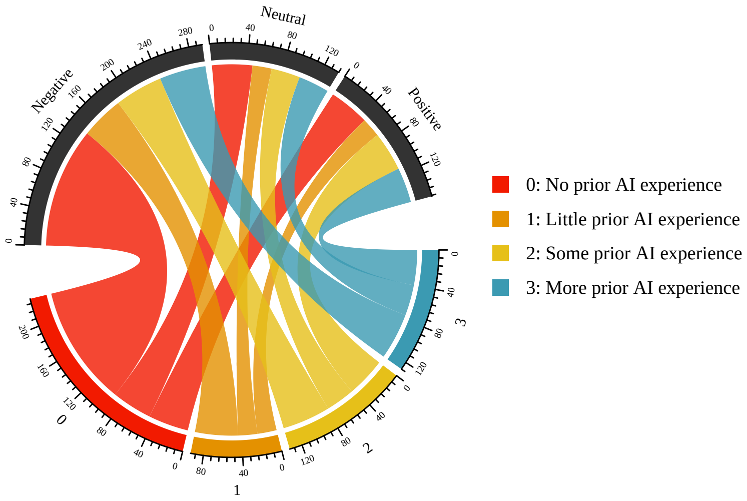

S4 Fig. Respondent sentiments on bias in algorithms, by level of prior experience with AI.

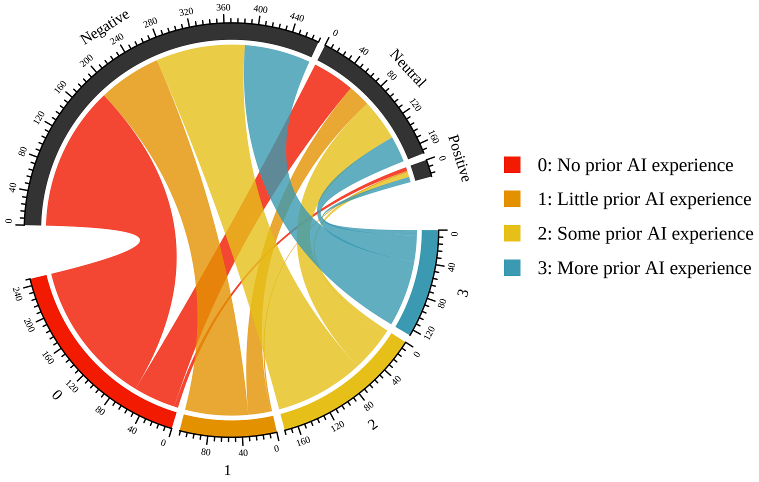

S5 Fig. Respondent sentiments on autonomous vehicles, by gender.

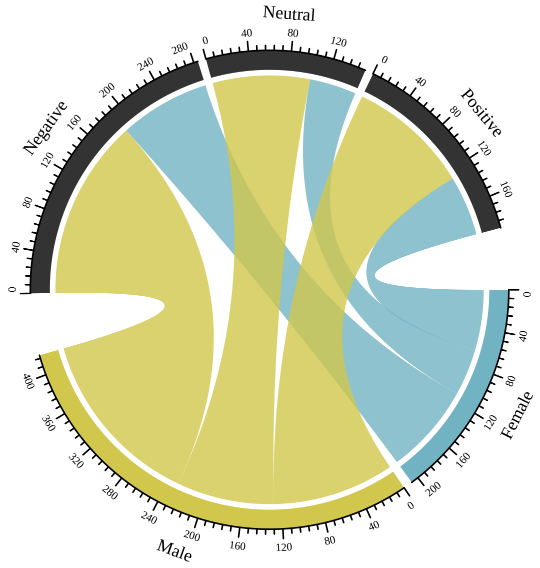

S6 Fig. Respondent sentiments on autonomous surgery, by gender.

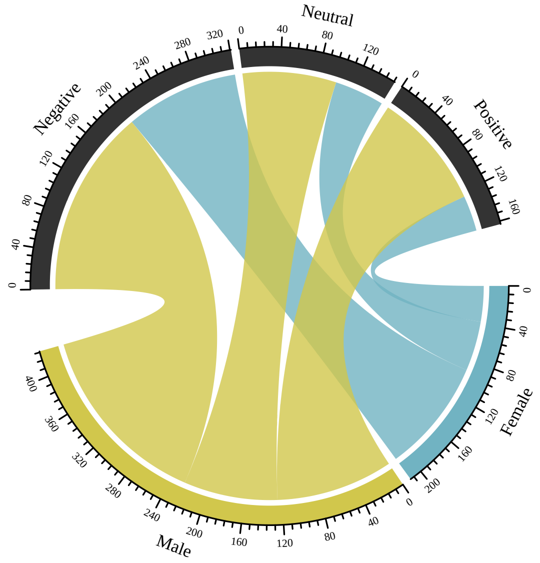

S1 Table: Drivers of Attitudes about Autonomous Vehicles and Autonomous Surgery.

	(1)<br>Autonomous Vehicles<br>OLS<br>b/SE	(2)<br>Autonomous Surgery<br>OLS<br>b/SE
Female	-0.333***	-0.536***
	(0.115)	(0.123)
Age	0.078***	-0.011
	(0.026)	(0.031)
White	-0.114	-0.158
	(0.180)	(0.232)
Education: 1 = No HS, 7 = Grad Degree	0.175***	0.153***
	(0.036)	(0.048)
Party ID: 1=Dem, 2=Ind, 3=GOP	-0.150***	-0.069
	(0.055)	(0.078)
Top 10 Auto Manufacturing State	-0.383*	
	(0.194)	
Health Care Employment By State		0.001
		(0.009)
Pre-COVID Rideshare App Usage	0.344***	
	(0.088)	
Self-Reported AI Use	0.235***	0.165***
	(0.039)	(0.049)
Support Info Gathering Over Privacy	-0.120**	-0.284***
	(0.057)	(0.072)
Concern about Algorithmic Bias	-0.151**	-0.049
	(0.063)	(0.073)
Urban Population	0.146**	-0.148*
	(0.059)	(0.082)
College Educated Population	-0.073	0.139*
	(0.077)	(0.078)
Unemployment Rate	-0.141***	0.003
	(0.051)	(0.069)
Constant	2.000***	2.877***
	(0.505)	(0.542)
Observations	465	462
R^2	0.311	0.201
Log Likelihood	-688.349	-710.112
F	38.977	9.501

Notes: Standard errors clustered by state in parentheses. *p<0.10; **p< 0.05; ***p<0.01.

S2 Table: Drivers of Attitudes about Uses of AI.

	(1)<br>Facial Recognition<br>Software<br>OLS<br>b/SE	(2)<br>General<br>Monitoring<br>OLS<br>b/SE	(3)<br>State<br>Jobs<br>OLS<br>b/SE	(4)<br>Prison<br>Sentences<br>OLS<br>b/SE	(5)<br>Transplant<br>List<br>OLS<br>b/SE	(6)<br>Natural Disaster<br>Impact Planning<br>OLS<br>b/SE	(7)<br>Responding To<br>911 Calls<br>OLS<br>b/SE	(8)<br>Military<br>Surveillance<br>OLS<br>b/SE	(9)<br>Use of<br>Military Force<br>OLS<br>b/SE
Female	-0.089	0.035	-0.164	-0.048	-0.088	-0.224*	-0.464***	-0.100	-0.247***
	(0.113)	(0.135)	(0.135)	(0.112)	(0.100)	(0.130)	(0.128)	(0.109)	(0.092)
Age	0.054**	0.012	0.040	0.061**	0.073**	0.028	0.072**	0.056*	-0.003
	(0.025)	(0.026)	(0.029)	(0.025)	(0.032)	(0.026)	(0.028)	(0.031)	(0.028)
White	0.137	0.144	-0.032	0.156	0.164	-0.090	-0.263	0.141	-0.132
	(0.129)	(0.145)	(0.196)	(0.153)	(0.153)	(0.183)	(0.161)	(0.155)	(0.202)
Education: 1 = No HS, 7 = Grad Degree	0.016	-0.023	-0.016	-0.061**	-0.049	0.042	0.038	0.034	-0.011
	(0.034)	(0.033)	(0.034)	(0.025)	(0.034)	(0.029)	(0.030)	(0.035)	(0.031)
Party ID: 1=Dem, 2=Ind, 3=GOP	0.208***	0.089	0.023	-0.033	-0.105**	-0.087	-0.094*	0.077	0.226***
	(0.049)	(0.057)	(0.046)	(0.065)	(0.051)	(0.057)	(0.050)	(0.046)	(0.055)
Pre-COVID Rideshare App Usage	-0.052	-0.054	-0.065	-0.084	-0.072	0.083	-0.106	-0.042	-0.075
	(0.086)	(0.069)	(0.090)	(0.062)	(0.079)	(0.053)	(0.076)	(0.049)	(0.054)
Self-Reported AI Use	0.030	0.036	0.089*	0.088	0.136***	0.108**	0.142***	0.119***	0.050
	(0.052)	(0.043)	(0.046)	(0.053)	(0.047)	(0.044)	(0.051)	(0.042)	(0.050)
Support Info Gathering Over Privacy	-0.245***	-0.320***	-0.160***	-0.235***	-0.147**	-0.087	-0.140*	-0.144**	-0.184***
	(0.053)	(0.074)	(0.050)	(0.045)	(0.056)	(0.056)	(0.078)	(0.062)	(0.056)
Concern about Algorithmic Bias	-0.148***	-0.192***	-0.158***	-0.109**	-0.156***	-0.149***	-0.106**	-0.160***	-0.191***
	(0.041)	(0.052)	(0.050)	(0.041)	(0.048)	(0.048)	(0.043)	(0.054)	(0.043)
Urban Population	-0.107	-0.031	-0.079	-0.025	0.073	-0.083	-0.029	0.032	0.064
	(0.072)	(0.065)	(0.064)	(0.069)	(0.087)	(0.088)	(0.070)	(0.073)	(0.073)
College Educated Population	-0.009	-0.070	0.023	-0.030	0.152***	0.153**	0.098	-0.073	0.020
	(0.071)	(0.052)	(0.071)	(0.058)	(0.049)	(0.060)	(0.066)	(0.052)	(0.076)
Unemployment Rate	0.117	0.051	0.019	-0.000	-0.109*	0.041	-0.032	0.108	0.073
	(0.072)	(0.071)	(0.069)	(0.053)	(0.063)	(0.060)	(0.094)	(0.075)	(0.060)
Constant	2.375***	2.699***	2.488***	2.305***	2.368***	3.077***	2.745***	2.550***	2.583***
	(0.372)	(0.346)	(0.473)	(0.422)	(0.384)	(0.393)	(0.460)	(0.440)	(0.364)
Observations	445	438	419	420	407	439	437	430	419
R^2	0.234	0.272	0.145	0.195	0.193	0.139	0.147	0.164	0.169
Log Likelihood	-583.123	-563.467	-547.372	-535.707	-523.441	-575.188	-638.674	-574.294	-590.862
F	37.404	16.767	11.317	12.212	17.848	7.719	20.459	16.706	13.112

Notes: Standard errors clustered by state in parentheses. *p<0.10; **p< 0.05; ***p<0.01.

S3 Table: Drivers of Attitudes about Uses of Autonomous Vehicles and Autonomous Surgery: Smaller Sample.

	(1)<br>Autonomous Vehicles<br>OLS<br>b/SE	(2)<br>Autonomous Surgery<br>OLS<br>b/SE
Female	-0.343***	-0.497***
	(0.112)	(0.132)
Age	0.087***	0.007
	(0.028)	(0.028)
White	-0.212	-0.088
	(0.185)	(0.210)
Education: 1 = No HS, 7 = Grad Degree	0.197***	0.163***
	(0.032)	(0.048)
Party ID: 1=Dem, 2=Ind, 3=GOP	-0.117*	-0.172*
	(0.061)	(0.101)
Top 10 Auto Manufacturing State	-0.468**	
	(0.190)	
Health Care Employment By State		0.002
		(0.008)
Pre-COVID Rideshare App Usage	0.387***	
	(0.092)	
Self-Reported AI Use	0.223***	0.147**
	(0.045)	(0.061)
Support Info Gathering Over Privacy	-0.146**	-0.253**
	(0.059)	(0.095)
Concern about Algorithmic Bias	-0.152*	-0.056
	(0.081)	(0.097)
Urban Population	0.125*	-0.115
	(0.068)	(0.102)
College Educated Population	-0.107	0.092
	(0.098)	(0.094)
Unemployment Rate	-0.155*	-0.002
	(0.078)	(0.085)
Constant	1.906***	2.783***
	(0.598)	(0.549)
Observations	329	328
R^2	0.348	0.210
Log Likelihood	-475.451	-495.638
F	39.392	9.875

Notes: Standard errors clustered by state in parentheses. *p<0.10; **p< 0.05; ***p<0.01.

S4 Table: Drivers of Attitudes about Autonomous Vehicles and Autonomous Surgery: No Probability Weights.

	(1) Self-Driving Cars OLS b/SE	(2) Autonomous Surgery OLS b/SE
Female	-0.308***	-0.461***
	(0.108)	(0.124)
Age	0.083***	-0.000
	(0.026)	(0.025)
White	0.038	0.016
	(0.173)	(0.177)
Education: 1 = No HS, 7 = Grad Degree	0.140***	0.129***
	(0.031)	(0.036)
Party ID: 1=Dem, 2=Ind, 3=GOP	-0.142**	-0.080
	(0.058)	(0.059)
Top 10 Auto Manufacturing State	-0.315*	
	(0.158)	
Health Care Employment By State		0.006
		(0.007)
Pre-COVID Rideshare App Usage	0.354***	
	(0.081)	
Self-Reported AI Use	0.220***	0.158***
	(0.038)	(0.038)
Support Info Gathering Over Privacy	-0.112**	-0.285***
	(0.053)	(0.054)
Concern about Algorithmic Bias	-0.084	-0.051
	(0.057)	(0.062)
Urban Population	0.136**	-0.122
	(0.052)	(0.073)
College Educated Population	0.022	0.120
	(0.070)	(0.075)
Unemployment Rate	-0.055	0.040
	(0.053)	(0.053)
Constant	1.546***	2.480***
	(0.438)	(0.523)
Observations	465	462
R^2	0.258	0.180
Log Likelihood	-698.862	-703.653
F	28.171	10.957

Notes: Standard errors clustered by state in parentheses. *p<0.10; **p< 0.05; ***p<0.01.

S5

Table: Drivers of Attitudes about Uses of AI: No Probability Weights.

	(1) Facial Recognition Software OLS b/SE	(2) General Monitoring OLS b/SE	(3) State Jobs OLS b/SE	(4) Prison Sentences OLS b/SE	(5) Transplant List OLS b/SE	(6) Natural Disaster Impact Planning OLS b/SE	(7) Responding To 911 Calls OLS b/SE	(8) Military Surveillance OLS b/SE	(9) Use of Military Force OLS b/SE
Female	-0.184**	-0.002	-0.106	-0.110	-0.047	-0.211**	-0.444***	-0.096	-0.097
	(0.085)	(0.097)	(0.100)	(0.084)	(0.087)	(0.102)	(0.112)	(0.090)	(0.090)
Age	0.063***	0.019	0.042**	0.062***	0.079***	0.035*	0.071***	0.066**	0.032
	(0.017)	(0.016)	(0.019)	(0.019)	(0.025)	(0.019)	(0.020)	(0.025)	(0.023)
White	0.042	0.039	0.046	0.087	0.100	0.021	-0.143	0.190	-0.166
	(0.132)	(0.114)	(0.150)	(0.118)	(0.141)	(0.147)	(0.112)	(0.158)	(0.164)
Education: 1 = No HS, 7 = Grad Degree	-0.006	-0.045	-0.037	-0.079***	-0.057*	0.035	0.043	0.019	-0.034
	(0.035)	(0.030)	(0.029)	(0.026)	(0.032)	(0.030)	(0.026)	(0.034)	(0.030)
Party ID: 1=Dem, 2=Ind, 3=GOP	0.247***	0.074	-0.015	-0.026	-0.104**	-0.068	-0.068	0.124***	0.240***
	(0.046)	(0.047)	(0.040)	(0.052)	(0.043)	(0.044)	(0.041)	(0.044)	(0.051)
Pre-COVID Rideshare App Usage	-0.062	-0.024	-0.049	-0.068	-0.032	0.085*	-0.026	-0.020	-0.034
	(0.078)	(0.062)	(0.076)	(0.058)	(0.069)	(0.046)	(0.053)	(0.049)	(0.050)
Self-Reported AI Use	0.029	0.010	0.077**	0.071*	0.110**	0.100**	0.116***	0.117**	0.041
	(0.045)	(0.042)	(0.035)	(0.038)	(0.041)	(0.040)	(0.042)	(0.044)	(0.043)
Support Info Gathering Over Privacy	-0.318***	-0.372***	-0.169***	-0.250***	-0.135***	-0.106**	-0.157***	-0.186***	-0.206***
	(0.041)	(0.049)	(0.035)	(0.038)	(0.044)	(0.047)	(0.058)	(0.051)	(0.051)
Concern about Algorithmic Bias	-0.091**	-0.171***	-0.135***	-0.095**	-0.160***	-0.088**	-0.103**	-0.096*	-0.176***
	(0.036)	(0.036)	(0.042)	(0.041)	(0.044)	(0.034)	(0.042)	(0.052)	(0.037)
Urban Population	-0.095	0.024	-0.038	0.003	0.090	-0.062	-0.013	-0.012	0.085
	(0.066)	(0.057)	(0.051)	(0.057)	(0.078)	(0.071)	(0.057)	(0.058)	(0.069)
College Educated Population	-0.033	-0.075	-0.026	0.020	0.142***	0.165***	0.069	-0.060	0.050
	(0.071)	(0.046)	(0.069)	(0.055)	(0.048)	(0.057)	(0.068)	(0.053)	(0.070)
Unemployment Rate	0.091	0.036	-0.007	-0.037	-0.126**	0.076	-0.016	0.130**	0.114**
	(0.061)	(0.053)	(0.064)	(0.045)	(0.057)	(0.047)	(0.079)	(0.063)	(0.051)
Constant	2.364***	2.792***	2.531***	2.360***	2.377***	2.667***	2.485***	2.211***	2.155***
	(0.325)	(0.280)	(0.359)	(0.295)	(0.314)	(0.284)	(0.331)	(0.416)	(0.319)
Observations	445	438	419	420	407	439	437	430	419
R^2	0.279	0.299	0.135	0.188	0.183	0.114	0.135	0.167	0.183
Log Likelihood	-573.193	-547.139	-539.198	-534.161	-525.469	-567.200	-628.772	-573.829	-592.097
F	44.145	21.831	14.707	14.216	19.417	10.740	16.502	17.078	12.015

Notes: Standard errors clustered by state in parentheses. *p<0.10; **p< 0.05; ***p<0.01.

S6 Table: Drivers of Attitudes about Uses of Autonomous Vehicles and Autonomous Surgery: Smaller Sample: No Probability Weights.

	(1) Self-Driving Cars OLS b/SE	(2) Autonomous Surgery OLS b/SE
Female	-0.270**	-0.409***
	(0.107)	(0.134)
Age	0.093***	0.018
	(0.027)	(0.025)
White	-0.074	0.007
	(0.185)	(0.188)
Education: 1 = No HS, 7 = Grad Degree	0.167***	0.147***
	(0.032)	(0.039)
Party ID: 1=Dem, 2=Ind, 3=GOP	-0.126*	-0.158**
	(0.066)	(0.074)
Top 10 Auto Manufacturing State	-0.441**	
	(0.168)	
Health Care Employment By State		0.007
		(0.007)
Pre-COVID Rideshare App Usage	0.340***	
	(0.096)	
Self-Reported AI Use	0.233***	0.144***
	(0.045)	(0.050)
Support Info Gathering Over Privacy	-0.138**	-0.301***
	(0.057)	(0.064)
Concern about Algorithmic Bias	-0.084	-0.030
	(0.074)	(0.082)
Urban Population	0.122	-0.084
	(0.076)	(0.091)
College Educated Population	0.012	0.097
	(0.093)	(0.091)
Unemployment Rate	-0.035	0.031
	(0.077)	(0.066)
Constant	1.469***	2.282***
	(0.457)	(0.545)
Observations	329	328
R^2	0.280	0.197
Log Likelihood	-488.455	-491.784
F	30.323	8.673

Notes: Standard errors clustered by state in parentheses. *p<0.10; **p< 0.05; ***p<0.01.

S7 Table: Drivers of Attitudes about Uses of Autonomous Vehicles and Autonomous Surgery: Smaller Sample: Ordinal Logit.

	(1) Self-Driving Cars OLS b/SE	(2) Autonomous Surgery OLS b/SE
Female	-0.579***	-0.870***
	(0.203)	(0.215)
Age	0.137***	-0.029
	(0.045)	(0.056)
White	-0.136	-0.237
	(0.311)	(0.405)
Education: 1 = No HS, 7 = Grad Degree	0.310***	0.236***
	(0.065)	(0.078)
Party ID: 1=Dem, 2=Ind, 3=GOP	-0.249**	-0.142
	(0.097)	(0.124)
Top 10 Auto Manufacturing State	-0.606*	
	(0.312)	
Health Care Employment By State		-0.001
		(0.014)
Pre-COVID Rideshare App Usage	0.645***	
	(0.167)	
Self-Reported AI Use	0.405***	0.265***
	(0.064)	(0.081)
Support Info Gathering Over Privacy	-0.217**	-0.517***
	(0.109)	(0.121)
Concern about Algorithmic Bias	-0.242**	-0.056
	(0.114)	(0.120)
Urban Population	0.238**	-0.241*
	(0.104)	(0.144)
College Educated Population	-0.144	0.193
	(0.139)	(0.126)
Unemployment Rate	-0.219**	0.021
	(0.090)	(0.124)
cut1	0.169	-1.912**
	(0.885)	(0.913)
cut2	1.137	-0.873
	(0.875)	(0.907)
cut3	2.449***	0.179
	(0.870)	(0.928)
cut4	4.640***	2.518***
	(0.863)	(0.897)
Observations	465	462
Pseudo R^2	0.120	0.076
Log Likelihood	-623.086	-642.227
Chi Squared	349.850	108.707

Notes: Standard errors clustered by state in parentheses. *p<0.10; **p< 0.05; ***p<0.01.

S8 Table: Drivers of Attitudes about Uses of AI: Ordinal Logit.

	(1) Facial Recognition Software OLS b/SE	(2) General Monitoring OLS b/SE	(3) State Jobs OLS b/SE	(4) Prison Sentences OLS b/SE	(5) Transplant List OLS b/SE	(6) Natural Disaster Impact Planning OLS b/SE	(7) Responding To 911 Calls OLS b/SE	(8) Military Surveillance OLS b/SE	(9) Use of Military Force OLS b/SE
main									
Female	-0.169	0.084	-0.271	-0.082	-0.187	-0.462	-0.802***	-0.245	-0.484***
	(0.236)	(0.275)	(0.286)	(0.250)	(0.220)	(0.282)	(0.217)	(0.202)	(0.172)
Age	0.102**	0.033	0.092	0.137**	0.160**	0.060	0.127**	0.110*	0.000
	(0.052)	(0.059)	(0.064)	(0.062)	(0.073)	(0.054)	(0.053)	(0.057)	(0.056)
White	0.272	0.430	-0.065	0.215	0.321	-0.111	-0.409	0.293	-0.329
	(0.264)	(0.422)	(0.441)	(0.380)	(0.346)	(0.381)	(0.254)	(0.282)	(0.387)
Education: 1 = No HS, 7 = Grad Degree	0.021	-0.048	-0.041	-0.118**	-0.090	0.097*	0.058	0.046	-0.009
	(0.075)	(0.073)	(0.074)	(0.053)	(0.072)	(0.059)	(0.057)	(0.073)	(0.056)
Party ID: 1=Dem, 2=Ind, 3=GOP	0.454***	0.172	0.037	-0.096	-0.197*	-0.175	-0.147	0.159*	0.438***
	(0.106)	(0.122)	(0.098)	(0.138)	(0.104)	(0.130)	(0.091)	(0.094)	(0.101)
Pre-COVID Rideshare App Usage	-0.050	-0.125	-0.091	-0.177	-0.111	0.113	-0.165	-0.098	-0.127
	(0.176)	(0.167)	(0.216)	(0.170)	(0.177)	(0.113)	(0.134)	(0.092)	(0.118)
Self-Reported AI Use	0.064	0.110	0.162*	0.181	0.299***	0.266**	0.252***	0.242**	0.108
	(0.108)	(0.088)	(0.090)	(0.114)	(0.094)	(0.108)	(0.091)	(0.097)	(0.091)
Support Info Gathering Over Privacy	-0.560***	-0.764***	-0.325***	-0.486***	-0.327***	-0.189	-0.248*	-0.319***	-0.383***
	(0.109)	(0.189)	(0.101)	(0.107)	(0.117)	(0.120)	(0.138)	(0.102)	(0.112)
Concern about Algorithmic Bias	-0.293***	-0.401***	-0.336***	-0.259***	-0.320***	-0.284***	-0.179**	-0.348***	-0.375***
	(0.085)	(0.107)	(0.098)	(0.086)	(0.103)	(0.107)	(0.084)	(0.106)	(0.083)
Urban Population	-0.259*	-0.064	-0.125	-0.072	0.163	-0.179	-0.059	0.033	0.121
	(0.152)	(0.146)	(0.134)	(0.142)	(0.181)	(0.191)	(0.121)	(0.149)	(0.144)
College Educated Population	0.004	-0.183	0.085	-0.068	0.309***	0.338***	0.184	-0.139	0.030
	(0.146)	(0.127)	(0.156)	(0.118)	(0.107)	(0.112)	(0.117)	(0.107)	(0.143)
Unemployment Rate	0.255*	0.138	0.057	0.037	-0.191	0.045	-0.044	0.248	0.143
	(0.141)	(0.172)	(0.148)	(0.114)	(0.135)	(0.116)	(0.166)	(0.152)	(0.117)
/									
cut1	-1.133	-1.722**	-1.334	-1.228	-1.028	-2.258**	-1.299	-1.558	-1.301*
	(0.744)	(0.733)	(1.019)	(1.026)	(0.806)	(0.880)	(0.815)	(0.948)	(0.684)
cut2	-0.027	-0.581	-0.114	0.060	0.391	-1.259	-0.524	-0.673	-0.454
	(0.755)	(0.725)	(1.062)	(1.004)	(0.856)	(0.820)	(0.830)	(0.869)	(0.694)
cut3	2.027**	1.521*	2.074*	2.219**	2.535***	0.676	1.042	1.207	1.310*
	(0.808)	(0.782)	(1.112)	(0.968)	(0.885)	(0.866)	(0.889)	(0.887)	(0.718)
Observations	445	438	419	420	407	439	437	430	419
Pseudo R^2	0.101	0.131	0.061	0.086	0.081	0.061	0.057	0.077	0.075
Log Likelihood	-517.743	-466.607	-484.670	-465.923	-475.272	-502.466	-545.356	-490.956	-489.778
Chi Squared	296.949	138.248	89.251	105.720	176.244	85.163	170.097	157.140	89.660

Notes: Standard errors clustered by state in parentheses. *p<0.10; **p< 0.05; ***p<0.01.

S9 Table: Drivers of Attitudes about Uses of Autonomous Vehicles and Autonomous Surgery: Smaller Sample: Ordinal Logit.

	(1) Self-Driving Cars OLS b/SE	(2) Autonomous Surgery OLS b/SE
Female	-0.582***	-0.858***
	(0.213)	(0.245)
Age	0.154***	-0.003
	(0.056)	(0.053)
White	-0.259	-0.093
	(0.310)	(0.369)
Education: 1 = No HS, 7 = Grad Degree	0.373***	0.270***
	(0.060)	(0.085)
Party ID: 1=Dem, 2=Ind, 3=GOP	-0.210*	-0.327*
	(0.114)	(0.167)
Top 10 Auto Manufacturing State	-0.733**	
	(0.330)	
Health Care Employment By State		-0.002
		(0.012)
Pre-COVID Rideshare App Usage	0.732***	
	(0.184)	
Self-Reported AI Use	0.431***	0.255**
	(0.084)	(0.101)
Support Info Gathering Over Privacy	-0.264**	-0.484***
	(0.119)	(0.175)
Concern about Algorithmic Bias	-0.276*	-0.068
	(0.151)	(0.168)
Urban Population	0.197*	-0.224
	(0.117)	(0.179)
College Educated Population	-0.181	0.110
	(0.177)	(0.160)
Unemployment Rate	-0.245*	0.003
	(0.134)	(0.152)
cut1	0.406	-2.034**
	(1.082)	(0.912)
cut2	1.380	-0.927
	(1.075)	(0.872)
cut3	2.777***	0.227
	(1.060)	(0.874)
cut4	5.061***	2.605***
	(1.109)	(0.870)
Observations	329	328
Pseudo R^2	0.138	0.082
Log Likelihood	-419.401	-443.033
Chi Squared	342.930	105.603

Notes: Standard errors clustered by state in parentheses. *p<0.10; **p< 0.05; ***p<0.01.
