# Supplementary material for: What influences attitudes about artificial intelligence adoption: Evidence from U.S. local officials
Source: PLoS One. 2021 Oct 20;16(10):e0257732. doi: 10.1371/journal.pone.0257732 (PMC8528275; doi:10.1371/journal.pone.0257732)
Supplement: S2 File — (PDF) [file pone.0257732.s002.pdf]

S2 Variable Definitions.

We include the following independent variables in our regression models, which we defined here.

- Sex (1 = Female, 0 = Male)
- Age (Continuous)
- Level of education (1 = No HS, 7 = Grad Degree)
- Political party (1 = Democrat/Lean Democrat, 2 = Independent, 3 = Republican/Lean Republican)
- Use of Ridesharing Apps (pre-COVID) (0 = never, 5 = almost every day or more)
- Does respondent live in top 10 Auto Manufacturing State (Vehicles model only) (1 = yes, 0 = no)
- Percentage of employment from hospitals (Surgery model only)
- Self-Reported AI use (0 = No prior self-reported AI experience, 3 = More prior self-reported AI experience)
- Relative support for information gathering versus privacy protection (1 = Strongly support gaining information over the risk to privacy, 5 = Strongly support protecting privacy over gaining information)
- Level of concern about algorithmic bias (1 = Not at all concerned, 5 = Very concerned)
