# Supplementary material for: What influences attitudes about artificial intelligence adoption: Evidence from U.S. local officials
Source: PLoS One. 2021 Oct 20;16(10):e0257732. doi: 10.1371/journal.pone.0257732 (PMC8528275; doi:10.1371/journal.pone.0257732)
Supplement: S3 File — (PDF) [file pone.0257732.s003.pdf]

S3 Survey Text.

Vehicles1
Prior to the COVID-19 pandemic, how frequently did you use ridesharing apps such as Lyft and Uber?

- Never
- A few times a year
- A few times a month
- A few times a week
- Almost every day or more
- I do not know what ridesharing apps are

Vehicles2
Imagine that automotive technology has advanced to the point where self-driving cars and trucks require little or no input from humans to operate. Would you support or oppose this development?

- Strongly support
- Somewhat support
- Neither support nor oppose
- Somewhat oppose
- Strongly oppose

Vehicles3
Please explain why you answered the way you did about self-driving cars.
Vehicles4
How concerned are you about the safety of autonomous vehicles for those riding in them as well as for other vehicles, cyclists, and pedestrians?

- Not at all concerned
- Slightly concerned
- Moderately concerned
- Very concerned
- Extremely concerned

Vehicles5
How likely would you be to ride in an autonomous vehicle?

- Very likely
- Somewhat likely
- Neither more nor less likely
- Somewhat unlikely
- Very unlikely

Vehicles6
Since the beginning of the COVID-19 pandemic, have you become more supportive or less supportive of autonomous vehicles?

- Much more supportive
- Somewhat more supportive
- Neither more nor less supportive
- Somewhat less supportive
- Much less supportive

Healthcare1
Imagine that medical technology has advanced to the point where surgical procedures will be able to be performed by autonomous systems (systems trained by an algorithm) with little to no input from humans. Would you support or oppose this development?

- Strongly support
- Somewhat support
- Neither support nor oppose
- Somewhat oppose
- Strongly oppose

Healthcare2
Please explain why you answered the way you did about autonomous systems surgery.

Healthcare3
How concerned are you about the safety of procedures for patients who undergo surgery conducted by autonomous systems?

- Not at all concerned
- Slightly concerned
- Moderately concerned

- Very concerned
- Extremely concerned

Healthcare4
Would you support or oppose the use of artificial intelligence to make decisions about the allocation of limited health care resources?

- Strongly support
- Somewhat support
- Neither support nor oppose
- Somewhat oppose
- Strongly oppose

Healthcare5
Since the beginning of the COVID-19 pandemic, have you become more supportive or less supportive of autonomous surgery.

- Much more supportive
- Somewhat more supportive
- Neither more nor less supportive
- Somewhat less supportive
- Much less supportive

AIgrid1 through Aigrid9
How do you feel about the possible use of algorithms or artificial intelligence in the following areas? [Order randomly presented]

1. Surveillance of criminal suspects through facial recognition software and other means
2. General monitoring of the civilian population for illicit or illegal behavior
3. Job selection and promotion for local officials
4. Decisions about prison sentences
5. Decisions about the transplant list
6. Natural disaster impact planning
7. Responding to 911 calls
8. Surveillance and monitoring of military targets
9. Use of military force

Response options:

- Very Unsupportive
- Somewhat Unsupportive
- Somewhat Supportive
- Very Supportive
- No Opinion

General1
In thinking about adopting the use of algorithms, how should governments balance the potential to gain useful information that could improve public safety with the potential to violate people's individual privacy?

- Strongly support gaining information over the risk to privacy
- Somewhat support gaining information over the risk to privacy
- Equally important
- Somewhat support protecting privacy over gaining information
- Strongly support protecting privacy over gaining information

General2
How concerned are you about the potential for bias in algorithms?

- Not at all concerned
- Slightly concerned
- Moderately concerned
- Very concerned
- Extremely concerned

General3
Please explain, in your own words, why you answered the way you did about the potential for bias in algorithms.
AIHome-Work
Generally speaking, do you use artificial intelligence at work or at home?

- At home
- At work
- At work and at home
- Neither

AIMusic-Movies
Do you use artificial intelligence-based systems to select music or movies for your enjoyment (e.g. Pandora or Netflix)?

- Yes
- No
