# Supplementary material for: What influences attitudes about artificial intelligence adoption: Evidence from U.S. local officials
Source: PLoS One. 2021 Oct 20;16(10):e0257732. doi: 10.1371/journal.pone.0257732 (PMC8528275; doi:10.1371/journal.pone.0257732)
Supplement: S4 File — (PDF) [file pone.0257732.s004.pdf]

S4 Text Analysis Coding Scheme.

Each text response should be applicable to at least 1 category, and up to a maximum of 3. If you feel that more than 3 categories apply, select the top 3 prominent/most relevant ones.

These categories are all in relations to sentiment about self-driving cars/surgery/AI in general (i.e. is this sentiment positive, negative, or neutral about AI).

Human Safety

- Accounts for human error, accidents
- Positive = commentary on how AI will be better for human safety, compared to human drivers
- Negative = commentary on how AI will be worse for human safety, compared to human drivers

Machine Safety

- Accounts for machine error, accidents, hacking
- Positive = commentary on how AI application makes accidents/failures less likely
- Negative = commentary on how AI application makes accidents/failures more likely

Control

- Accounts for responses that express sentiments related to control, such as a desire to be in control, unwillingness to relinquish control, or happiness to delegate control.
- Positive = commentary on willingness/happiness to delegate control
- Negative = expressions of concern about delegating control

Personal Experience

- Accounts for responses that highlight there is some personal background, experience, or knowledge to their response.
- This includes they have worked on the topic before, know someone who does, are a tech expert etc.
- Positive = expressing how knowledge/personal experience -> trust AI application
- Negative = expression how knowledge/personal experience -> do not trust AI application

Humanity/Morality

- Accounts for responses that highlight things that should be uniquely restricted to humans, or that are uniquely human experiences.
- This could included responses such as individuals prefer driving themselves, think those making medical decisions should be humans, worried about judgement calls/ethical decisions, thinks more technology can distance.
- Positive = expressing how AI application is more moral, unbiased than humans, help humans, etc.
- Negative = expressing how the task the AI is completing should be a human endeavor, judgements made by AI won't be appropriate for humans

Technical Reliability

- Focused on the actual tech itself, how good/bad it is, unreliable/reliable, how well it works etc.
- Positive = trust AI application because it is technically reliable, work well, are advanced, make technology more efficient, etc.
- Negative = do not trust AI application because it is technically unreliable, do not work well, etc.

Societal

- Focused on societal benefits/harms i.e. it will help those who cannot typically drive themselves, it will hurt taxi drivers/harm jobs. Also questions of efficiency in society.
- Positive = AI application will help those who cannot previously do this task, fill a need in society, for example self-driving cars will allow disabled people to typically drive themselves
- Negative = negative impact on jobs from self-driving cars

Political/Regulatory/Legal

- Focused on political or regulation or legal aspects
- Positive = Discussion of how political/regulatory/legal issues will make AI application be positive
- Negative = Discussion of how political/regulatory/legal issues will make AI application be negative

Implementation

- Focused on actual practicality/feasibility of use/implementation economically/financially, if it will be tested enough, if it can be used in specific contexts/states/roads etc.

- Positive = the AI application could be implemented with a reasonable amount of effort; example: self-driving cars are inexpensive and will work with existing roads
- Negative = the AI application is not a good fit for a specific context/"juice is not worth the squeeze" example: self-driving cars are expensive and don't work with existing roads

Polarity:

- Positive
- Neutral
- Negative
